# Introgressed mitochondrial fragments from archaic hominins alter nuclear genome function in modern humans

**DOI:** 10.1126/sciadv.aea0706

**Published:** 2026-02-04

**Authors:** Qiong Zhu, Jinning Zhang, Weichen Zhou, Shen-Ao Liang, Shengmiao Wang, Xinyu Cai, Fuyuan Li, Jin Li, Guojie Zhang, Huijuan Feng, Qiaomei Fu, Joshua M. Akey, Feng Zhang, Li Jin, Shuhua Xu, Hong-Xiang Zheng, Lu Chen

**Affiliations:** ^1^ State Key Laboratory of Genetics and Development of Complex Phenotypes, Center for Evolutionary Biology, School of Life Science, Fudan University, Shanghai 200438, China.; ^2^ Department of Computational Medicine and Bioinformatics, University of Michigan Medical School, Ann Arbor, MI 48109, USA.; ^3^ Department of Life Sciences, Imperial College London, London SW7 2AZ, UK.; ^4^ Department of Cell and Development Biology, State Key Laboratory of Genetics and Development of Complex Phenotypes and School of Life Sciences, Fudan University, Shanghai 200433, China.; ^5^ Center for Evolutionary and Organismal Biology, Liangzhu Laboratory, Zhejiang University School of Medicine, Hangzhou, China.; ^6^ Department of Computational Biology, School of Life Science, Fudan University, Shanghai 200438, China.; ^7^ Key Laboratory of Vertebrate Evolution and Human Origins, Institute of Vertebrate Paleontology and Paleoanthropology, Chinese Academy of Sciences, Beijing 100044, China.; ^8^ University of the Chinese Academy of Sciences, Beijing 100049, China.; ^9^ The Lewis-Sigler Institute for Integrative Genomics, Princeton University, Princeton, NJ 08540, USA.; ^10^ School of Life Science and Technology, ShanghaiTech University, Shanghai 201210, China.

## Abstract

Archaic introgression introduced functionally relevant variants into modern humans, yet small-scale insertions remain understudied. Here, we leverage 2519 modern human genomes and four high-coverage archaic hominin genomes to systematically characterize nuclear mitochondrial DNA segments (NUMTs). We uncover 483 polymorphic NUMTs across globally diverse human populations and 10 in archaic genomes. By combining overlap with Neanderthal-derived and Denisovan-derived haplotypes, phylogenetic analyses, insertion time estimates, and haplotype colocalization, we identify five NUMTs introduced into modern humans via archaic hominin introgression. Functional analyses reveal that introgressed NUMTs can modulate gene expression, including allele-specific up-regulation of the immune-related gene *RASGRP3*, and reshape three-dimensional chromatin structure at loci such as *SCD5* and *HNRNPD*. These findings highlight an underappreciated mechanism by which archaic mitochondrial fragments shape nuclear genome function and evolution. Our study reframes NUMTs not as passive genomic fossils but as dynamic elements influencing modern human diversity and adaptation.

## Teaser

Nuclear mitochondrial DNA from archaic hominins is uncovered in humans and shown to affect gene regulation and genome structure.

## INTRODUCTION

Archaic introgression has substantially shaped the genetic and phenotypic diversity of modern human populations by introducing functionally relevant variants into the genomes (*1*–*3*). Although extensive studies have focused predominantly on large-scale introgressed segments (typically >30 kb) (*4*), there remain many classes of potentially functional archaic variants that are still underexplored. In particular, small-scale insertions derived from archaic hominins are often overlooked, leading to an incomplete understanding of archaic genetic legacies and their evolutionary impacts on modern humans.

One prominent but understudied class of small-scale insertions is nuclear mitochondrial DNA segments (NUMTs)—fragments of mitochondrial DNA (mtDNA) integrated into the nuclear genome. NUMTs represent an ongoing evolutionary phenomenon and have been widely documented across diverse eukaryotic genomes, including humans (*5*–*12*). Previous research has identified hundreds of polymorphic NUMTs in modern humans (*13*–*16*), which range in size from a few base pairs to the full mitochondrial genome, revealing their potential roles in shaping genomic structure and influencing human health (*9*). Functionally, NUMTs have been implicated in genetic disorders (*14*, *17*–*19*), particularly when their insertions disrupt genes (*15*), and they can also confound mitochondrial genetic assays (*20*, *21*).

Despite their recognized importance in modern humans (*7*, *22*), NUMTs in archaic hominin genomes remain poorly studied. Historically, this research gap is largely attributed to technical limitations arising from low-coverage ancient DNA data (*23*). However, recent advancements in sequencing technologies have provided high-quality genomic data from archaic hominins such as Neanderthals and Denisovans (*24*–*27*), facilitating more comprehensive investigations in archaic NUMTs. Notably, a recent report identified a Denisovan-derived NUMT in an Oceanian individual, highlighting the possibility that NUMTs could be transferred to modern human populations through archaic admixture events (*24*).

Here, we systematically investigate the landscape of NUMTs in both archaic and modern human genomes. Using high-coverage genomic data from three Neanderthals, one Denisovan, and 2519 globally diverse modern individuals, we identify and characterize NUMTs of potential archaic origin, quantify their overlap with known introgressed genomic segments, estimate insertion times, and assess their functional implications. Our results expand the current understanding of archaic genetic introgression, revealing NUMTs as a previously overlooked category of archaic variants with notable evolutionary and functional relevance in modern human populations.

## RESULTS

### NUMT landscape across modern populations

**Fig. 1.**
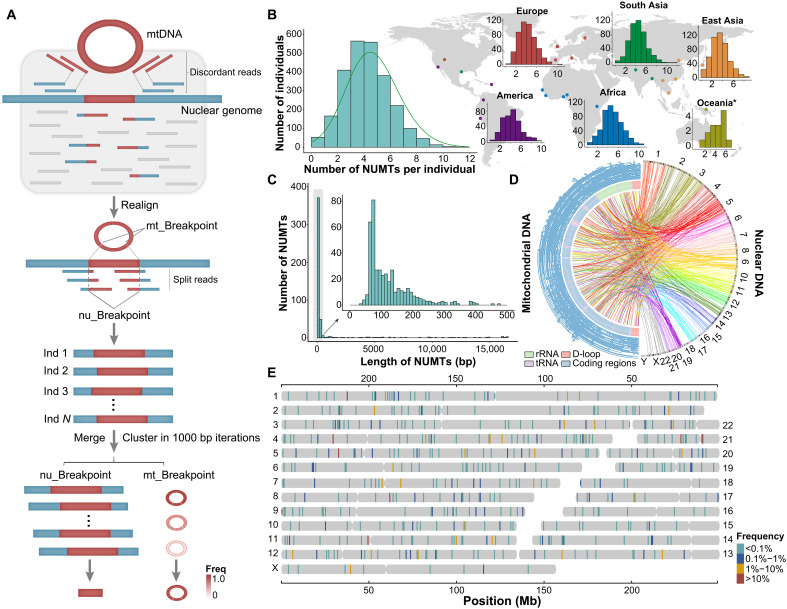
Detection and characterization of NUMTs in 2519 individuals. (**A**) Workflow for identifying NUMTs absent from the human reference genome, including the merging strategy across individuals. Nuclear breakpoints were defined as the first and second start positions of insertions within the cluster, whereas mitochondrial breakpoints were assigned on the basis of the most frequently observed positions within the cluster. (**B**) Average number of NUMTs per individual that are absent from the reference genome across all samples (turquoise) and stratified by population: America (purple), Europe (red), Africa (blue), South Asia (green), East Asia (orange), and Oceania (olive green). For the population-specific histograms, the axes are the same as in the histogram of all samples (*x* axis, number of NUMTs per individual; *y* axis, number of individuals). Note that the Oceanian group includes only 15 Papuan individuals. (**C**) Distribution of NUMT lengths. The inset highlights NUMTs shorter than 500 bp. (**D**) Circos plot showing 483 distinct NUMTs. The outer circle (light blue) represents mtDNA fragments corresponding to NUMTs; the inner circle denotes mitochondrial genes. Lines connect each mtDNA fragment to its insertion site in the nuclear genome. rRNA, ribosomal RNA. (**E**) Chromosomal locations of NUMT insertions, colored by their population frequency.

To investigate NUMTs in archaic lineages and their potential role in archaic introgression, we first characterized the landscape of NUMTs across globally diverse modern human populations. We applied a validated detection pipeline (*14*) to high-coverage whole-genome sequencing data of 2519 unrelated individuals from the 1000 Genomes Project (1KGP) (*28*) and the Simons Genome Diversity Project (SGDP) (*29*) (tables S1 and S2). For the analysis of Denisovan introgression, we specifically included Papuan individuals from SGDP. Nuclear insertion breakpoints were merged across individuals using an established clustering strategy (*29*) based on a 1000–base pair (bp) window, whereas mitochondrial breakpoints were defined as the most frequently observed positions through iterative clustering (see Materials and Methods and Fig. 1A).

In total, we identified 483 distinct NUMTs that are not present in the human reference genome (*30*), detected in 2509 individuals (99.6%), each supported by at least two discordant read pairs (table S3). Among these, 410 NUMTs were population specific, most of which were singletons, although a subset occurred at high frequencies within individual populations (table S4). In comparison with NUMTs detected in nonhuman primates, including chimpanzees, bonobos, gorillas, orangutans, and macaques (*31*), all NUMTs identified in our datasets are human specific. However, it is worth noting that this inference is based on limited comparative genomic data, and the absence of orthologous insertions in other primates may, in part, reflect incomplete sampling or limited detection sensitivity, as has been observed for other variants initially presumed to be human specific (*32*). When compared to a previous NUMT callset of 1KGP data based on an alternative detection pipeline (*33*), ~88% of NUMTs were consistently identified, with ~12% uniquely detected by our method (fig. S1 and table S3). To understand these discrepancies, we systematically examined unmatched loci (see Materials and Methods). Many sites missed by our pipeline showed insufficient read support upon manual inspection or failed to meet our more stringent breakpoint thresholds (fig. S2). Conversely, several NUMTs uniquely detected in our dataset were validated by long-read 1KGP samples (*34*), supporting their reliability (fig. S3 and table S5). These differences likely reflect variation in filtering criteria and read-mapping strategies across studies, with our approach prioritizing conservative, high-confidence calls.

On average, each individual carried ~4.7 NUMTs (SD = 1.8; Fig. 1B), consistent with previous studies (*13*, *14*). In addition, we observed significant population-level variation in NUMT counts (two-tailed *t* test; table S6). Notably, East Asians exhibit a distinct distribution compared to other populations, suggesting a role for population structure in shaping NUMT profiles (Fig. 1B and table S6). The lengths of NUMTs ranged from 39 bp to the entire mitochondrial genome (mean = 1071 bp; median = 111 bp; SD = 3177 bp), with the majority being short insertions: 76.8% were under 200 bp and 87.4% under 500 bp (Fig. 1C). The genomic insertion sites and mitochondrial coverage of these NUMTs are illustrated in Fig. 1D.

We further classified NUMTs based on population frequency: prevalent (*F* > 10%), common (1% ≤ *F* ≤ 10%), rare (0.1% ≤ *F* < 1%), and ultrarare (*F* < 0.1%). As expected, most NUMTs were ultrarare (73.7%), occurring in just 14.8% of individuals; 17.4% were rare (in 25.1% of individuals), 7.02% were common (in 54.3% of individuals), and only 1.85% were prevalent (in 98.9% of individuals) (Fig. 1E). Notably, of the 34 common NUMTs, 20 (59%) were predominantly found in the African populations, with each detected in at least 61% of African individuals.

### Identification of NUMTs in archaic genomes

We next examined NUMTs in archaic hominin lineages using four high-coverage genomes: three Neanderthals and one Denisovan. Given the highly degraded nature of ancient DNA, sequencing libraries from archaic remains typically consist of short DNA fragments (*35*), which often results in an insufficient number of split reads and poses technical challenges for reliable NUMT detection. To address this, we modified the calling strategy used for modern human genomes by lowering the threshold for the number of supporting split reads (see Materials and Methods). Using this adjusted approach, we identified five distinct NUMTs—four in Neanderthals and four in the Denisovan genome (fig. S4).

To mitigate potential reference bias introduced by modern mitochondrial genomes, we repeated the analysis using archaic mtDNA as alternative references, which enabled the detection of two additional NUMTs (fig. S5, A to C). Furthermore, we manually inspected candidate insertion breakpoints using visualization tools to improve confidence in NUMT identification in low-complexity ancient DNA data (fig. S5, D to F). To further rule out artifacts in archaic NUMTs, we leveraged NUMTs that are also observed in modern individuals for whom trio data are available and applied trio-based inheritance checks in modern carriers. Five of such cases were found, and in each case, the same NUMT insertion breakpoint is observed in the offspring and in one or both parents, demonstrating inheritance consistency and further confirming the authenticity of these germline insertions (fig. S6, A to E). This provides an orthogonal line of support that complements the archaic data.

In total, we identified 10 distinct NUMTs absent from the human reference genome across the four high-coverage archaic genomes: two in the Vindija Neanderthal, six in the Chagyrskaya Neanderthal, and seven in the Altai Denisovan (Table 1 and fig. S6F). No NUMTs were detected in the Altai Neanderthal genome, likely due to its greater antiquity [~130 thousand years ago (ka)] (*25*), resulting in insufficient coverage at candidate NUMT breakpoints. On average, each archaic genome harbored ~3.75 NUMTs that are not present in the reference genome. All archaic NUMTs were short insertions, with lengths below 600 bp (mean = 163 bp; median = 101 bp; SD = 155 bp). Notably, 7 of the 10 NUMTs (70%) were also found in modern human populations, with observed frequencies ranging from 0.08 to 66.61% (Table 1).

**Table. 1.** NUMTs identified in four archaic genomes and their frequencies across modern human populations. Note: nu_breakpoint indicates the breakpoint of NUMTs in the nuclear genome, whereas mt_breakpoint indicates the breakpoint in the mtDNA. 95% CI, 95% confidence interval.

nu_breakpoint	mt_breakpoint	NUMT length (bp)	Archaic genomes	Estimated age (95% CI) (ka)	Number of modern humans with NUMTs					
					EUR (503)^*^	AFR (504)^*^	AMR (347)^*^	EAS (504)^*^	SAS (489)^*^	Papua (15)^*^
chr1:88,592,028–88,592,037	4524–4686	162	Denisovan	–	0	0	0	0	0	0
chr2:146,676,955–146,676,963	7954–8034	80	Denisovan	217 (16, 524)	0	0	0	0	0	0
chr3:142,815,571–142,815,572	1378–1688	310	Denisovan/Chagyrskaya	427 (126, 746)	0	0	0	0	0	3
chr4:82,695,010–82,695,012	11,022–11,188	166	Denisovan	2571 (780, 4534)	6	29	5	1	14	1
chr4:178,496,733–178,496,733	5976–6018	42	Chagyryskaya	–	0	0	0	2	0	0
chr5:28,004,248–28,004,250	2824–2917	93	Denisovan/Chagyrskaya	304 (64, 591)	0	0	0	0	0	0
chr9:85,809,246–85,809,253	1410–1473	63	Chagyryskaya/Vindija	–	0	15	1	0	0	0
chr11:49,862,017–49,862,019	16,088–61	542	Vindija/Denisovan/Chagyrskaya	1488 (599, 2650)	392	198	275	377	335	9
chr11:100,145,004–100,145,018	11,498–11,557	59	Chagyrskaya	–	42	0	24	8	30	3
chr21:21,708,297–21,708,298	5712–5820	108	Denisovan	–	122	145	95	162	127	12

* Numbers in parentheses indicate the total number of individuals in each population.

### NUMTs embedded in Neanderthal segments

Notably, we identified several NUMTs that are shared between archaic and modern humans and persist at relatively high frequencies in present-day populations—for example, NUMT *chr11_49M* (66.6%) and *chr21_21M* (27.9%). At the same time, certain NUMTs identified in archaic genomes appear to be restricted to specific modern populations; for instance, NUMT *chr3_142M* was exclusively observed in Papuans at a frequency of 20%. This substantial proportion of NUMTs shared between modern and archaic genomes may be explained by three scenarios: (i) independent insertions in archaic and modern lineages (i.e., recurrent de novo events); (ii) a single insertion event in a common ancestor before lineage divergence, with persistence through shared ancestry or incomplete lineage sorting (ILS); and (iii) introgression of NUMTs from archaic hominins into modern humans via admixture. In addition, the complex history of gene flow among Neanderthals, Denisovans, and modern humans may have further facilitated such sharing (*36*–*38*). These scenarios are not mutually exclusive and may have occurred in a staggered, sequential manner over evolutionary time, although it also remains possible that only one of them actually took place.

To evaluate the contribution of archaic introgression to NUMT presence in modern genomes, we examined the colocalization of NUMTs and archaic introgressed segments on a per-individual basis. Given the short length of NUMTs, we reasoned that true introgressed NUMTs would be physically embedded within longer archaic-derived haplotypes. Because non-African modern humans harbor ~2% Neanderthal ancestry—substantially higher than Denisovan ancestry levels (*39*)—we initially focused our analysis on Neanderthal introgressed sequences. For each individual, we assessed the overlap between previously identified Neanderthal introgressed segments (*36*) and NUMTs detected in the same genome.

Despite the short length and relatively low number of NUMTs per genome, we observed that 22 distinct NUMTs (4.6%) overlapped with Neanderthal introgressed segments within the same individual genome in 219 modern genomes (Table 2). Some overlapping NUMTs (e.g., NUMTs *chr11_100M* and *chr11_49M*) occurred at relatively high frequencies across multiple populations, whereas the majority were rare and sparsely distributed. Five of the NUMTs that overlapped with Neanderthal segments were also detected in archaic genomes, representing 50% (5/10) of all archaic NUMTs identified. Among these five, four (80%) were common (frequency > 1%) and widely distributed across modern populations. Notably, *chr11_49M*—the most frequent NUMT in modern humans—was among them (Table 1). To validate these findings, we repeated the overlap analysis using two additional callsets of Neanderthal introgressed segments identified by alternative approaches, S* and Sprime, and observed consistent results (table S7).

**Table. 2.** NUMT insertions overlapping Neanderthal introgressed segments in 1KGP individuals.

nu_breakpoint	mt_breakpoint	Numbers of individuals with NUMTs overlapping Neanderthal segments					
		Total	EUR	AFR	AMR	SAS	EAS
chr1:170,256,486–170,256,486	9801–10,192	4	0	0	0	4	0
chr1:37,611,748–37,611,748	8933–9007	17	8	0	1	6	2
chr1:59,195,879–59,195,879	15,420–15,478	4	0	4	0	0	0
chr1:61,649,661–61,649,662	5958–6076	1	0	0	0	1	0
chr11:100,145,004–100,145,004^*^	11,498–11,557	91	39	0	18	28	6
chr11:49,862,017–49,862,017^*^	16,088–61	52	34	1	9	8	0
chr11:99,188,486–99,188,490	9421–9487	1	0	0	0	1	0
chr12:87,959,136–87,959,136	6483–6550	1	0	0	0	1	0
chr15:51,673,831–51,673,832	4960–5631	1	0	0	0	0	1
chr2:33,667,411–33,667,411	14,776–15,022	24	5	0	5	11	3
chr2:34,418,573–34,418,876	5700–5820	1	0	0	0	1	0
chr2:68,801,196–68,801,206	10,191–16,397	1	1	0	0	0	0
chr2:79,899,154–79,899,154	7283–7381	1	0	0	1	0	0
chr21:21,708,297–21,708,297^*^	5712–5820	1	0	0	0	1	0
chr3:17,999,761–17,999,761	12,288–13,613	1	0	0	0	0	1
chr4:126,419,043–126,419,043	14,030–14,098	9	0	0	2	0	7
chr4:178,496,733–178,496,733^*^	5976–6018	1	0	0	0	0	1
chr4:82,695,010–82,695,010^*^	11,022–11,188	3	1	1	1	0	0
chr5:32,338,477–32,338,477	14,830–12,714	7	5	0	1	1	0
chr6:102,877,808–102,877,808	14,641–14,844	1	0	0	0	0	1
chr6:68,179,572–68,179,572	7010–2699	2	0	0	0	2	0
chr8:62,102,863–62,102,863	7083–8553	1	0	1	0	0	0

* NUMTs were present in archaic samples.

### Evidence of Neanderthal introgressed NUMTs

**Fig. 2.**
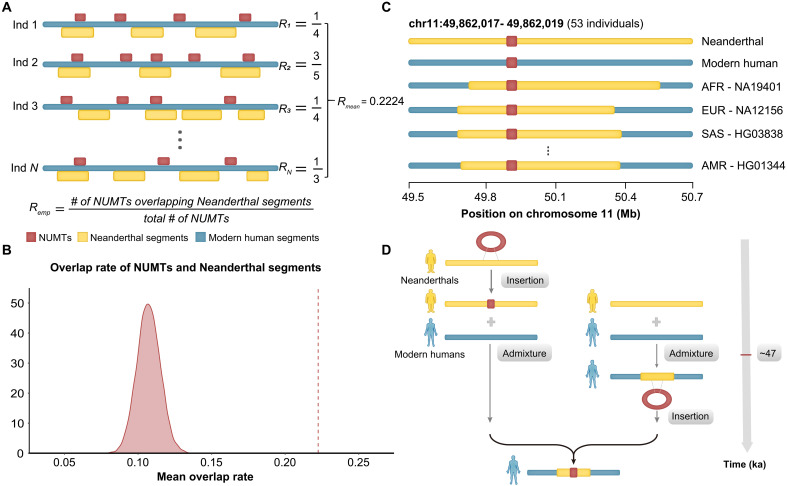
NUMTs are significantly enriched with Neanderthal introgressed segments in 219 modern individuals. (**A**) Schematic illustrating how an enrichment of NUMTs and Neanderthal introgressed segments was analyzed. For each individual, the rate of overlap between NUMTs and Neanderthal segments was calculated, and the mean across these 219 individuals was taken as the empirical value. (**B**) Null distribution of mean overlap rates from permutation test, with the empirical value shown as a dashed line. (**C**) Representative example of an NUMT (*chr11_49M*) colocalized with a Neanderthal introgressed region across modern human genomes. Red indicates NUMT fragments, yellow represents Neanderthal segments, and blue denotes modern human sequences. (**D**) Two hypothetical scenarios explaining NUMT-Neanderthal segment colocalization in modern genomes. Left: An NUMT inserts into the Neanderthal genome and is later introduced into modern humans via admixture. Right: Following the Neanderthal–modern human admixture, the NUMT insertion occurs within a Neanderthal derived segment in a modern human individual.

To investigate whether the observed colocalization of NUMTs and Neanderthal introgressed segments in modern individuals reflects genuine introgression rather than random coincidence or other processes, we performed a permutation-based statistical test across 219 individuals (see Materials and Methods). For each individual, we calculated the proportion of NUMTs that overlapped with Neanderthal introgressed regions (Fig. 2A). We then averaged these rates of overlap to calculate the empirical genome-wide rate of overlap among these individuals. Notably, we found the rate of overlap with Neanderthal introgressed segments to be highly significant (*P* < 0.0002, permutation test) (Fig. 2B), supporting the hypothesis that certain NUMTs were introduced into the modern human genome via archaic admixture rather than by chance. We replicated this finding using two alternative sets of Neanderthal introgressed sequences identified by S* (*40*) and Sprime (*41*) methods and obtained consistent results (fig. S7). Together, these data show one line of evidence for introgression, and these 22 NUMTs overlapping introgressed regions might be potential candidates for putative Neanderthal introgressed NUMTs. Nonetheless, additional evidence is required to establish introgression as their definitive origin.

To disentangle possible evolutionary explanations for such NUMTs, exemplified by *chr11_49M* (Fig. 2C), whose presence overlaps Neanderthal derived segments in modern genomes—including ILS, post–admixture insertion into archaic haplotypes (hitchhiking) (Fig. 2D), and true introgression—we integrated multiple lines of evidence. We first estimated the insertion times for three NUMTs among the 22 candidates. Because of the limited length and sequence divergence of most NUMTs, we were only able to estimate insertion times for 3 of the 22 NUMTs. These NUMT insertions occurred at ~1488 ka (*chr11_49M*), 553 ka (*chr2_33M*), and 2571 ka (*chr4_82M*), each substantially predating the Neanderthal–modern human introgression event (~47 ka) (*42*). Notably, the two older insertions also predate the estimated divergence time between modern humans and Neanderthals. This temporal evidence can rule out recent insertions hitchhiking on introgressed haplotypes in these NUMTs.

Next, we examined overlap with multiple orthogonal Neanderthal introgression callsets (IBDmix using Altai, Vindija, and Chagyrskaya genomes; Sprime; and S*). All three NUMTs showed consistent overlap with Neanderthal tracts in at least two callsets. For example, *chr4_82M* colocalized with segments detected by both IBDmix and Sprime; *chr11_49M* was identified in IBDmix using Vindija and Chagyrskaya references; and *chr2_33M* was supported by both IBDmix and Sprime. These multi-callset signals reduce the likelihood of false positives due to reference biases.

To further test whether these overlapping signals reflect genuine introgression rather than chance colocalization, we examined phased haplotype data to assess whether NUMTs and introgressed segments are carried on the same chromosome (see Materials and Methods). For *chr2_33M* and *chr4_82M*, phased genotypes from the 1KGP variant cell format (VCF) file were combined with Sprime-inferred archaic segments. Among 12 heterozygous individuals carrying both *chr2_33M* and a Neanderthal segment, 5 showed colocalization on the same haplotype. For *chr4_82M*, individuals carrying introgressed segments were homozygous for the NUMT and heterozygous for the archaic segment, also consistent with coinheritance. For *chr11_49M*, although IBDmix tracts lack phasing, 52 individuals were homozygous for both the NUMT and the overlapping introgressed segment, strongly indicating shared haplotypes. Together with previous evidence, this analysis further confirms that the introgressed segment and NUMT insertion co-occur on the same chromosome, reinforcing an archaic origin rather than recurrent insertion or hitchhiking.

**Fig. 3.**
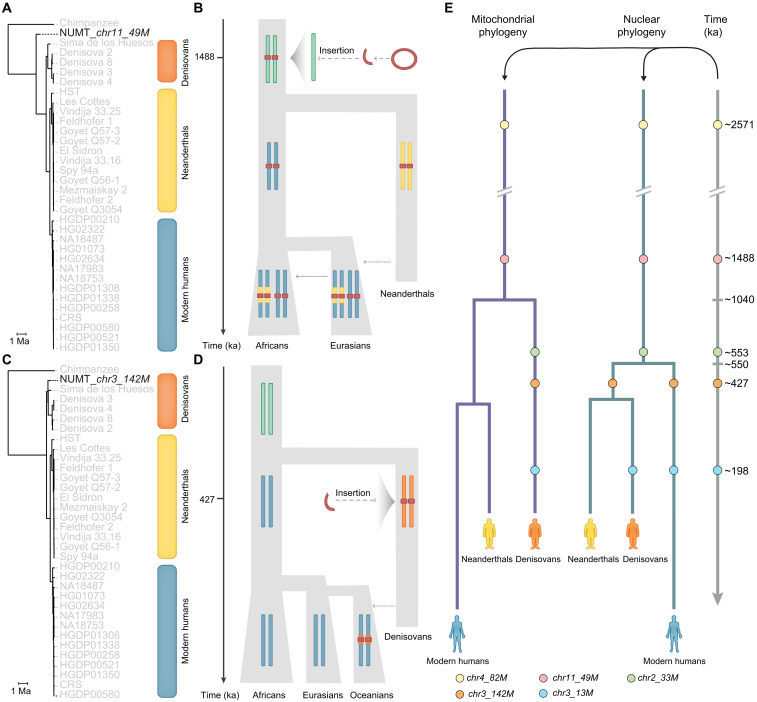
Insertion timing and evolutionary trajectories of introgressed NUMTs from archaic hominins. (**A**) Phylogenetic tree of NUMT *chr11_49M*, showing that it forms a sister clade to all Denisovans, Neanderthals, and modern humans. The orange block represents the Denisovan lineage, the yellow block represents Neanderthals, and the blue block represents modern humans. Ma, million years ago. (**B**) Schematic demographic model illustrating the evolutionary history of NUMT *chr11_49M*, originating ~1488 ka, followed by the divergence of archaic and modern human lineages and later introgression into modern humans. (**C**) Phylogenetic tree of NUMT *chr3_142M*, which clusters specifically within the Denisovan clade (orange block). (**D**) Schematic demographic model illustrating the evolutionary history of NUMT *chr3_142M*, which originated in the Denisovan lineage ~427 ka, followed by introgression into modern human genomes. (**E**) Phylogenetic placement of five introgressed NUMTs based on mitochondrial (left) and nuclear (right) phylogenies. Each colored circle represents an NUMT (e.g., *chr4_82M*), positioned according to its estimated insertion time and phylogenetic context. Vertical dashed lines mark key divergence points between archaic and modern human lineages.

We further analyzed phylogenetic relationships using mtDNA sequences corresponding to these NUMTs from various archaic and modern humans, with chimpanzee as an outgroup (Fig. 3A and fig. S8). The insertions of NUMT *chr11_49M* and *chr4_82M* took place after the divergence of humans and chimpanzees, whereas NUMT *chr2_33M* was inserted after the split between Denisovan and Neanderthal–modern human mitochondrial lineages. Moreover, *chr11_49M* and *chr4_82M* were also directly detected in at least one high-coverage archaic genome, providing orthogonal support of their deep origins. Intriguingly, for both loci, modern individuals fall into two haplotype categories: those carrying introgressed flanking sequences and those carrying nonintrogressed flanks, a pattern consistent with a mixture of ILS and introgression, but not with recent human insertions hitchhiking on Neanderthal haplotypes. A schematic of NUMT *chr11_49M* across hominin lineages illustrates this mixed ancestry scenario (Fig. 3B).

To rule out hotspot-driven recurrent insertions, we compared nuclear breakpoints and NUMT sequences across carriers (see Materials and Methods). All individuals shared identical nuclear breakpoints, and the reconstructed NUMT sequences were nearly identical (≥99.87% pairwise similarity; table S8). These findings indicate a single ancestral insertion rather than multiple independent insertions.

Collectively, these multiple lines of evidence—including significant statistical enrichment, orthogonal callset overlap, deep insertion time, phylogenetic clustering, archaic genome detection, shared breakpoints, and haplotype-level colocalization—strongly *chr4_82M*, *chr11_49M*, and *chr2_33M* represent genuine Neanderthal-introgressed NUMTs (or at least include introgressed alleles in modern populations). These results reveal a previously underappreciated mechanism of archaic mtDNA transmission through nuclear insertions, adding a distinct dimension to our understanding of mitochondrial-nuclear genome interactions.

### Denisovan introgressed NUMTs in Papuans

Building on the evidence for Neanderthal-derived NUMTs, we next investigated whether Denisovan introgression contributed similar insertions to present-day genomes. Given that Denisovan ancestry is low in 1KGP individuals but reaches up to ~5% in Papuan genomes (*43*), we leveraged high-coverage Papuan genomes from SGDP for this analysis. We identified two NUMTs (*chr3_142M* and *chr4_82M*) that overlap Denisovan introgressed segments in Papuans on a per-individual level (table S9). To further infer their evolutionary origins, we estimated insertion times for 16 NUMTs found in Papuans. Of these, five NUMTs (*chr3_13M*, *chr3_142M*, *chr4_82M*, *chr11_49M*, and *chr2_33M*) yielded reliable age estimates, ranging from ~198 ka to ~2571 ka. Notably, two NUMTs, *chr3_142M* (~427 ka) and *chr4_82M* (~2571 ka), that overlapped Denisovan introgressed segments exhibited insertion times predating the estimated Denisovan–modern human admixture event (~44 to 54 ka) (*39*), supporting an archaic origin. Moreover, both insertions were directly identified in the high-coverage Denisovan genome, further validating their presence in the archaic lineage before introgression.

Among these, *chr3_142M* represents the strongest candidate for Denisovan introgression. The NUMT is phylogenetically nested within the Denisovan mitochondrial clade (Fig. 3C). In addition, all three carriers of this NUMT in SGDP Papuans also harbor Denisovan introgressed haplotypes flanking the insertion site (Fig. 3D), and all carriers share identical nuclear breakpoints and 100% sequence identity (table S8), excluding the possibility of recurrent insertions at a hotspot. These multiple lines of evidence support a model in which the *chr3_142M* insertion occurred in the Denisovan lineage and was later transmitted to modern humans via introgression.

In addition, one of the NUMTs identified in Papuans, *chr3_13M*, was previously reported as part of a Denisovan introgressed haplotype (*24*). Although we did not observe a direct overlap between this NUMT and Denisovan segments in our dataset, the estimated insertion time (~198 ka) and phylogenetic placement within the Denisovan mtDNA clade (fig. S8) support point to a Denisovan origin. The absence of detected flanking introgression may reflect the small sample size of Papuans with this NUMT or limitations of current callsets.

These findings highlight a broader role for archaic admixture—from both Neanderthals and Denisovans—in shaping the modern human NUMT landscape. Collectively, they support a model in which NUMT insertions occurred either predating the split between archaic and modern human lineages or in archaic hominins and were later transmitted to modern humans via introgression. A conceptual summary of these dynamics is shown in Fig. 3E. A compact summary table of the five best-supported introgressed NUMTs with detailed information is shown in table S10.

### Positive selection of NUMTs

**Fig. 4.**
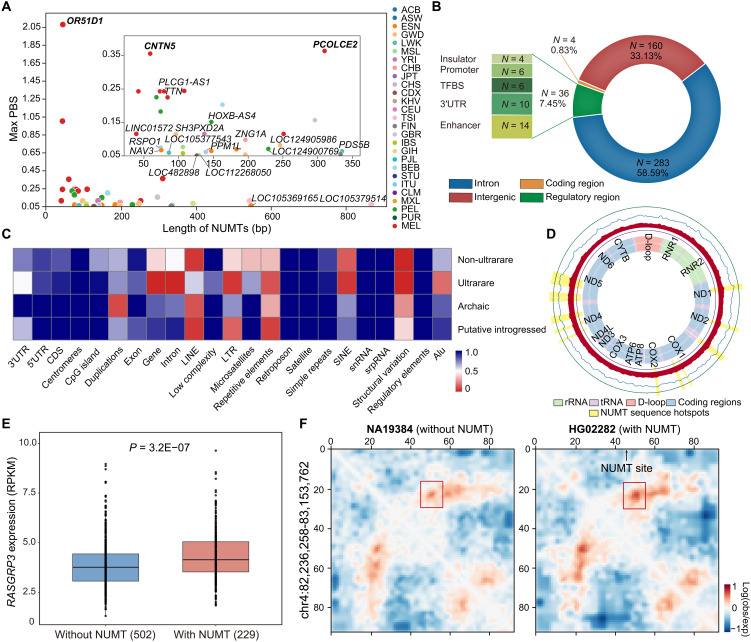
Genomic distribution and functional consequences of NUMTs in modern humans. (**A**) PBS values of NUMTs located within 5 kb of annotated genes. Each dot represents an NUMT, with the colors corresponding to populations. The bolded genes represent NUMT-adjacent loci with strong signals of population-specific selection (|*Z*| > 2). (**B**) Functional annotation of the 483 NUMTs. Inset table shows the number of NUMTs overlapping regulatory elements in noncoding regions. (**C**) *P* values from enrichment hotspot analysis across various genomic annotations. “Non-ultrarare” refers to NUMTs classified as rare, common, or prevalent (frequency ≥ 0.1%). The color scale represents *P* values from the enrichment analysis, with red indicating lower values (i.e., stronger enrichment) and blue indicating higher values (i.e., no enrichment). LTR, long terminal repeat; snRNA, small nuclear RNA; srpRNA, signal recognition particle RNA. (**D**) Mitochondrial origin distribution of NUMT fragments. From outer to inner tracks: (i) frequency of mtDNA breakpoints; (ii) average frequency of NUMT segments (50-bp sliding windows); (iii) frequency of mtDNA segments from NUMTs; (iv) average frequency from 1000 random simulations; and (v) reference mitochondrial genome with gene annotations. Yellow bars highlight significantly enriched mtDNA hotspot regions. (**E**) Expression of gene *RASGRP3* in individuals with (*n* = 229) and without (*n* = 502) the introgressed NUMT *chr2_33M*. *P* values were calculated by the Wilcoxon rank sum test. RPKM, reads per kilobase of transcript per million mapped reads. (**F**) Chromatin contact maps at the chr4:82,236,258–83,153,762 locus for NA19384 (without *chr4_82M*; left) and HG02282 (with *chr4_82M*; right) at a 10-kb resolution. The red box highlights the region showing altered contact frequencies after the insertion, and the arrow indicates the NUMT insertion site. The color scale indicates log(obs/exp) contact frequency.

The observation that certain NUMTs are maintained at high frequencies in modern human populations raises the possibility that they may have been subject to recent positive selection. To systematically test this hypothesis, we applied population branch statistics (PBS) (*44*) to detect population-specific frequency differentiation. We identified five NUMTs with elevated PBS values (>2 SDs; table S11), all of which are specifically enriched in Papuans (Fig. 4A). To ensure these signals were not driven by local genomic artifacts, we performed a block jackknife analysis by iteratively excluding genomic regions surrounding each NUMT (at 500-kb and 1-Mb resolution, respectively; see Materials and Methods). PBS signals remained robust across replicates, indicating they are not driven by single influential regions (table S11).

Three of these high-PBS NUMTs were previously associated with archaic introgression: one NUMT, *chr3_142M*, is confirmed as Denisovan derived, whereas NUMTs *chr11_100M* and *chr21_21M* were identified as putatively introgressed from Neanderthals (Table 2). Their high frequencies and population-specific differentiation suggest these insertions may have conferred adaptive benefits. To assess whether these frequency patterns reflect true selection rather than Papuan-specific population structure, we further applied the haplotype-based XP-EHH statistic (see Materials and Methods). NUMT *chr21_21M* showed a robust XP-EHH signal (>2) (table S11), providing orthogonal evidence for positive selection. The remaining four NUMTs did not exhibit significant XP-EHH values, which may reflect different characteristics between allele frequency-based and haplotype-based methods or limited power due to sample size.

Functionally, these positively selected NUMTs lie near genes with known biological relevance. For example, *chr3_142M* is located within 5 kb of the gene *PCOLCE2*, known to be involved in heparin binding and protease inhibition (*45*); *chr11_100M* directly inserts into the gene *CNTN5*, a gene essential for axon guidance and neural connectivity during development (*46*); and *chr21_21M* lies within 200 kb of the *NCAM2* gene, which encodes a neural cell adhesion molecule involved in the selective fasciculation and zone-to-zone projection of primary olfactory axons (*47*).

To further investigate potential selective pressures acting on archaic introgressed NUMTs, we analyzed the PBS distribution across all putative introgressed NUMTs and the confirmed introgressed NUMTs identified in our study. Thirty-six percent of these NUMTs ranked in the top 5% of PBS values relative to all identified NUMTs. Notably, among the five introgressed NUMTs with clear evolutionary trajectories, four (*chr3_142M*, *chr3_13M*, *chr11_49M*, and *chr2_33M*) showed PBS values within the highest 5% observed across all modern human NUMTs. These findings collectively support that archaic introgressed NUMTs provided adaptive advantages to modern human populations, likely mediated through regulatory interactions with nearby genes involved in critical biological pathways such as immunity and neural development.

### Characteristics of NUMT sequences

Motivated by the signals of positive selection detected among NUMTs, we sought to characterize their genomic distribution and investigate potential functional impacts. We first examined the sequence and insertional context of the 483 distinct NUMTs identified across modern human genomes, including both nuclear insertion sites and corresponding mtDNA fragments. NUMTs were more frequently inserted into introns (58.59%) than intergenic regions (33.13%), with four insertions located within coding regions (Fig. 4B). To gain deeper insights into their genomic associations, we systematically compared NUMT insertion sites with a range of annotated genomic elements, including coding sequences, untranslated regions (UTRs), centromeres, CpG islands, genomic duplications, retrotransposons [e.g., long interspersed nuclear elements (LINEs) and short interspersed nuclear elements (SINEs)], repetitive elements, and structural variations. NUMTs occurring at frequencies ≥ 0.1% (categorized as rare, common, or prevalent) were significantly enriched in proximity to structural variations (*P* = 0.001, two-tailed permutation test), whereas ultrarare NUMTs (*F* < 0.1%) exhibited preferential insertion into genes, introns, as well as structural variations (all *P* = 0.001). Notably, putative introgressed NUMTs showed specific enrichments near LINE elements (*P* = 0.024) and repetitive elements (*P* = 0.048), suggesting distinct insertion biases associated with introgression (Fig. 4C).

To further elucidate the mtDNA origins of NUMT fragments, we mapped NUMT sequences back to the mitochondrial genome using 50-bp sliding-window analysis. Our results revealed significant enrichment of NUMT-derived fragments within 12 distinct mitochondrial regions (*P* < 0.05, two-tailed permutation test), notably including segments of genes critical for mitochondrial function, such as *ND1*, *ND2*, *COX1*, and *COX2* (Fig. 4D). These findings indicate that NUMTs preferentially originate from specific mitochondrial genomic hotspots rather than through random integration events.

### Functional consequences of NUMT insertions

Given the demonstrated association of NUMTs with structural variations and regulatory elements, we next explored their functional consequences in terms of gene regulation. Using RNA sequencing (RNA-seq) data from lymphoblastoid cell lines (LCLs) of 731 individuals (*48*), we assessed whether introgressed or putative introgressed NUMTs could modulate gene expression at nearby loci (table S12). Among NUMTs present in sufficient frequency (≥10 carriers), three exhibited significant associations with altered expression patterns of nearby genes.

Notably, the confirmed introgressed NUMT *chr2_33M*, located ~100 kb upstream of *RASGRP3* (involved in immune signaling) (*49*) was strongly associated with increased expression of this gene (*P* = 3.2 × 10^−7^, Wilcoxon rank sum test; Fig. 4E). To evaluate whether this effect reflects cis-regulatory modulation by the NUMT haplotype, we further performed allele-specific expression analysis in 206 individuals heterozygous at this NUMT locus (see Materials and Methods). Using informative heterozygous exonic single-nucleotide polymorphisms (SNPs) in *RASGRP3*, we phased RNA-seq reads to compare allelic expression between haplotypes linked or unlinked to the NUMT. This analysis reveals a significant allelic imbalance favoring the NUMT-carrying haplotype (Wilcoxon signed-rank *P* = 7.88 × 10^−8^; fig. S9), supporting a cis-regulatory role.

Other putative introgressed NUMTs also showed regulatory associations: NUMT *chr5_32M* correlated with elevated expression of *ZFR*, a gene involved in RNA splicing (*50*) (*P* = 0.01); NUMT *chr1_37M* was associated with altered expression of *GNL2* (*P* = 2.2 × 10^−16^), *SNIP1* (*P* = 0.012), and *MEAF6* (*P* = 1.8 × 10^−10^), genes integral to transcriptional and chromatin regulatory processes (fig. S10, A to D). Notably, altered expression of *SNIP1* also correlated with increased expression of its target gene *CCND1*, a key regulator of the cell cycle (*51*) (*P* = 0.023; fig. S10E). These observations underscore the potential of archaic-origin NUMTs to affect transcriptional networks, influencing cellular and physiological processes.

Inspired by established evidence linking structural variants (SVs) with changes in three-dimensional (3D) genome architecture and gene regulation (*52*), we examined whether NUMT insertions similarly influence chromatin organization. Using Akita, a deep learning model that predicts 3D genome structure from primary DNA sequence (*53*), we identified NUMTs associated with substantial alterations in local chromatin interactions by using the mean squared error (MSE) between predicted contact matrices. Specifically, the confirmed introgressed NUMT *chr4_82M* (frequency = 6.6%), exhibiting an MSE within the upper 10th percentile relative to all NUMT insertions, showed marked alteration of chromatin contacts across a 200- to 300-kb region upstream of its insertion site (fig. S11A).

To compare with empirical chromatin structure, we analyzed Hi-C maps from HGSVC3 LCLs (see Materials and Methods). The *chr4_82M* carrier HG02282 exhibited a localized increase in contact frequency across the same upstream interval relative to the noncarrier NA19384 (Fig. 4F), potentially affecting the regulation of *SCD5* and *HNRNPD*, genes involved in energy metabolism (*54*) and mRNA regulation (*55*). Although the direction of effect differed from Akita predictions (increase versus predicted decrease), both datasets indicated substantial 3D architectural remodeling associated with the NUMT insertion. Similarly, we also observed that the putative introgressed NUMT *chr1_37M* (frequency = 29.5%), previously observed as association with differential expression of multiple nearby genes *GNL2*, *SNIP1*, and *MEAF6*, was supported by both Akita predictions and Hi-C evidence as perturbing local chromatin architecture (fig. S11, B and C).

Last, to complement these regulatory findings, we evaluated the coding potential of introgressed NUMTs by aligning 24 putative introgressed NUMTs (including five high-confidence ones) to the 13 mitochondrial protein-coding genes (*56*). Among high-confidence introgressed NUMTs, *chr4_82M* (*ND4* derived) harbors one synonymous and five nonsynonymous substitutions, and *chr2_33M* (*CYTB* derived) carries three synonymous and one nonsynonymous substitution (table S13). These observations point to the potential for some NUMTs to serve as novel coding elements within the nuclear genome.

Together, these findings provide preliminary yet converging evidence that NUMT insertions, particularly those of archaic origin, may act as previously underappreciated regulatory and functional elements that affect genome architecture and contribute to adaptive genomic innovations within the modern human genome (table S10).

## DISCUSSION

In this study, we systematically explored NUMTs in modern and archaic human genomes, revealing a previously underappreciated mechanism of archaic mtDNA transmission through nuclear insertions, adding a distinct dimension to our understanding of mitochondrial-nuclear genome interactions. By integrating high-coverage genomic datasets from diverse human populations and archaic hominins, we comprehensively analyzed the NUMTs we detected—by significant statistical enrichment, orthogonal callset overlap, deep insertion time, and phylogenetic clustering—and lastly identified multiple NUMTs as archaic introgressed NUMTs (or at least include introgressed alleles in modern populations): *chr4_82M*, *chr11_49M*, *chr2_33M*, *chr3_142M*, and *chr3_13M*.

However, detecting introgressed NUMTs presents unique methodological challenges. First, current introgression detection methods typically rely on identifying relatively long haplotypes to distinguish introgression from ILS, potentially overlooking smaller introgressed fragments that cannot be confidently identified and co-occur with NUMTs. Consequently, the actual proportion of introgressed NUMTs in modern populations is likely higher than our conservative estimates. Notably, the use of IBDmix, one of the introgression detection methods we used in this study, may also capture gene flow from modern humans to Neanderthals (*36*), raising the possibility that some NUMTs found in archaic genomes may have originated in early modern humans. Second, for some putative introgressed NUMTs, the lack of complete sequence reconstruction limits the availability of insertion time estimates or phylogenetic evidence. Future long-read sequencing will enable recovery of much longer NUMT sequences, allowing more accurate inference of insertion ages and more robust phylogenetic analyses.

Among these, NUMT *chr11_49M* stands out as a particularly compelling case. This NUMT is the most frequent insertion in modern humans and was also identified in Neanderthal genomes, with an estimated insertion time predating the divergence of archaic and modern humans. Intriguingly, this same NUMT was described over two decades ago as a mitochondrial D-loop “fossil” potentially informative about early human origins (*57*). Our findings build on this earlier work by confirming its Neanderthal origin and demonstrating its persistence through archaic introgression, highlighting its utility as a unique genetic marker that bridges ancient and modern genomes.

Two Denisovan-origin NUMTs—*chr3_142M* and *chr5_28M*—were detected in both the Altai Denisovan and Chagyrskaya Neanderthal genomes. Phylogenetic analyses confirm their clustering within the Denisovan lineage, and their estimated insertion times (~427 and ~304 ka, respectively) further support a Denisovan mitochondrial origin (Fig. 3C and fig. S12). A plausible explanation is that these NUMTs were inserted into the nuclear genome of early Neanderthals before the mitochondrial turnover event that replaced Denisovan-like mtDNA with an incoming lineage (*58*, *59*). In this scenario, the NUMTs effectively preserve a genetic snapshot of a pre-turnover mitochondrial state, subsequently fixed in the Neanderthal nuclear genome and retained independently of later mtDNA replacement. To our knowledge, this represents the genetic evidence of “pre-turnover” Denisovan-like mtDNA preserved in Neanderthals via NUMTs, offering a previously unrecognized window into deep mitochondrial history that would otherwise be erased by lineage replacement.

In addition to their evolutionary origins, our analyses suggest that a subset of introgressed NUMTs may have undergone population-specific selection. Several loci, particularly in Papuans, show elevated PBS, and one site (*chr21_21M*) is also supported by XP-EHH. These loci lie near genes involved in neurodevelopment and immune response, raising the possibility that introgressed NUMTs may have contributed to local adaptation via regulatory interactions. However, we caution that all PBS outliers were observed in Papuans, a population with complex demographic history—including more bottlenecks and deep divergence—that may inflate tail probabilities and confound outlier-based selection scans. For these reasons, combined with limited sample sizes, we view the selection evidence presented here as preliminary. Future studies will benefit from larger Papuan cohorts, coalescent simulations under realistic demographic histories, as well as long-read sequencing technologies, which provide more accurate haplotype resolution around NUMT insertions. Such data will allow improved detection of extended haplotype patterns, better phase inference, and more robust application of haplotype-based tests of recent positive selection.

Although NUMTs have traditionally been studied for their potential to disrupt coding regions, our analyses reveal that they can influence gene expression and chromatin organization through diverse regulatory mechanisms. For instance, the introgressed NUMT *chr2_33M* significantly increases expression of the immune-related gene *RASGRP3*, supported by allele-specific expression analysis indicating higher transcription from the NUMT-carrying haplotype. Although these findings provide preliminary support for cis-regulatory effects of introgressed NUMTs, replication in an independent cohort will be essential to strengthen these conclusions. In addition, by integrating deep learning–based predictions of 3D genome structure with empirical Hi-C maps, we identified NUMT insertions—such as the introgressed NUMT *chr4_82M*—that are associated with altered chromatin contact patterns and may affect the regulation of nearby genes like *SCD5* and *HNRNPD*. In some cases, the predicted and observed Hi-C effects were not fully concordant. We explicitly report this directional discordance and consider plausible explanations, including cell-type differences, model-training limitations, and the absence of NUMT-carrying haplotypes in training data. Together, these findings suggest a previously unrecognized regulatory role for NUMTs, paralleling known effects of structural variations on genome function and evolution, with requirements of further validation using additional datasets and targeted functional studies.

Collectively, our work redefines NUMTs as dynamic genomic elements, serving as both ancient genetic markers and active functional components of the modern human genome. These small-scale insertions, long regarded primarily as genomic fossils, emerge from our analyses as meaningful evolutionary players influencing chromatin organization, gene regulation, and adaptive processes. Future investigations using broader tissue-specific functional profiling and higher-resolution introgression mapping will further expand our understanding of NUMTs, enriching insights into human evolutionary biology and the complex interplay between nuclear and mitochondrial genomes.

## MATERIALS AND METHODS

### Data sources

The 2504 unrelated samples aligned to GRCh38 from the phase three panel of the 1KGP (*28*) can be accessed at the following link: https://internationalgenome.org/data-portal/data-collection/30x-grch38. The 15 Papua New Guinea samples aligned to GRCh38 from the SGDP (*29*) can be accessed at the following link: https://internationalgenome.org/data-portal/data-collection/sgdp. We downloaded FASTQ files for three previously published high-coverage archaic hominin genomes—a Denisovan (*60*) and a Neanderthal (*25*) from Denisova Cave in the Altai Mountain as well as a Neanderthal (*26*) from Vindija Cave. These files are available in the European Nucleotide Archive (ENA; ebi.ac.uk/ena) with study accession numbers: ERP001519, ERP002097, and PRJEB21157, respectively. The mitochondrial sequences assembly of Denisovan, Altai Neanderthal, Vindija Neanderthal, and Chagyrskaya Neanderthal were available in the GenBank database (https://ncbi.nlm.nih.gov/) (study accession numbers: NC_013993, KC879692, KJ533545, and MK388903, respectively). For the Neanderthal individual from Chagyrskaya Cave, only raw BAM files are available (http://ftp.eva.mpg.de/neandertal/Chagyrskaya/rawBAM).

Introgressed segments (*36*) identified by IBDmix were downloaded from: https://github.com/PrincetonUniversity/IBDmix/blob/main/IBDmix_calls_using_3_archaics.tar.gz. The Denisovan introgressed segments (*41*) identified in Papua New Guinea individuals using Sprime are available at: https://doi.org/10.17632/y7hyt83vxr.1. The RNA-seq BAM files for the 731 individuals in MAGE (*48*) were supplied by the Rajiv McCoy Lab. Hi-C data for LCLs from the Human Genome Structural Variation Consortium phase 3 (HGSVC3) are available at ftp.1000genomes.ebi.ac.uk/vol1/ftp/data_collections/HGSVC3/working/2021_Hi-C_JAX/. The 1019 long-read sequencing data can be downloaded via the following link: https://ftp.1000genomes.ebi.ac.uk/vol1/ftp/data_collections/1KG_ONT_VIENNA/hg38/.

### The processing of the archaic genome

To reduce the impact of uracil and ensure data integrity, for the Denisovan, Altai Neanderthal, and Vindija Neanderthal genomes, the first and last two bases of all reads in the downloaded raw FASTQ files were removed using fastp (*61*). Furthermore, because of the variations in the mitochondrial sequences among the Denisovan, Altai Neanderthal, and Vindija Neanderthal genomes, we substituted the mitochondrial sequences in GRCh38 with those from distinct archaic genomes, therefore producing five reference genomes with distinct mitochondrial sequences. Subsequently, the preprocessed reads were aligned to the five reference genomes using BWA-MEM (*62*), respectively, generating BAM files. Unmapped reads and PCR (polymerase chain reaction) duplicates were then excluded, and multiple libraries were merged to generate the final BAM file using SAMtools (*63*).

For the Chagyrskaya Neanderthal genome, single-end reads and paired-end reads within the BAM files were initially separated before being converted into FASTQ file format using SAMtools. The following processing of the Chagyrskaya Neanderthal genome was the same as the processing of other genomes.

### The identification and characterization of NUMTs

To detect NUMTs, we used a published and validated method (*14*). For phase three of the 1KGP, all discordant reads were initially extracted from the aligned CRAM files using samblaster (*64*). Reads with a mapping quality (MAPQ) equal to zero were excluded. Paired-end reads were then clustered on the basis of the orientation and distance within 500 bp, where one read aligned to mtDNA and the other to the nuclear genome. A minimum of two pairs of discordant reads was required for each cluster. To determine the exact breakpoints, all reads within the identified cluster regions were extracted and then realigned using BLAT (*65*). Further analysis was conducted on split reads where one end mapped to mtDNA and the other to the nuclear genome. Each NUMT needs to be supported by at least three split reads: one nuclear genome breakpoint and two mtDNA breakpoints. NUMTs in the reference genome and the breakpoints with local coverage exceeding 200x were excluded.

To merge NUMTs across different individuals, distinct strategies were used for nuclear genome breakpoints and mtDNA breakpoints. For nuclear genome breakpoints, similar to *dinumt* (*13*), breakpoints within 1000 bp were clustered. We determined the first and second start positions of the nuclear genome breakpoint as the start and end positions of the merged NUMTs’ nuclear genome breakpoint, respectively. As for mtDNA breakpoints, the frequencies of each mtDNA breakpoint within the cluster were calculated. The one with the highest frequency is defined as the merged NUMT’s mtDNA breakpoint. Last, we performed functional annotations using ANNOVAR (*66*).

For the four high-coverage archaic genomes, we used three different methods because the genomes contain paired-end and single-end reads and mtDNA reference bias. First, similar to modern human genomes, all discordant reads were extracted using samblaster and then clustered. To determine the exact breakpoints, all reads, including single-end and paired-end reads, were extracted from the clusters identified in the four archaic genomes and realigned using BLAT. Because of the lower coverage of archaic genomes, each NUMT breakpoint included one nuclear genome breakpoint and two mtDNA breakpoints which required at least one split read to support. Second, to reduce the effect of reference bias, we realigned the four archaic genomes with a reference genome replacing their respective mtDNA instead of modern human mtDNA. The following steps are the same as the steps used to detect NUMTs in modern humans. Third, to minimize omissions, we examined whether there are potential breakpoints of split reads around the NUMTs overlapped introgressed segments using IGV (*67*). Subsequently, we extended the NUMT region, extracted all reads from this area, realigned using BLAT, and identified the confirmed breakpoints. All archaic NUMTs identified through the three methods form the final archaic NUMTs call sets.

### Generation of NUMT sequences

For the nonreference NUMT, we extracted discordant reads and split reads from the read clusters and realigned them to the revised Cambridge Reference Sequence (rCRS) of the human mtDNA genome (*68*) using BWA-MEM using 1KGP sequencing data. The realigned mtDNA bam files were used to generate the sequence for each NUMT of an individual genome. First, the bases with a phred scaled quality of <10 were considered unknown low-quality bases and called *N*. Second, the alleles covered with less than three reads or the coverage frequency of the major allele less than 0.75 at heterozygous sites were called *N* (*69*). After generating the NUMT sequence for each individual, some sites showed different alleles as segregating sites among the 1KGP populations. Then, we used the major allele for each segregating site to construct the final representative NUMT sequence.

For the reference NUMTs, the coordinates in the GRCh38 reference genome were according to a previous study (*70*). Then, NUMT sequences were extracted on the basis of the coordinates and aligned to rCRS using blastn 2.14.1+ (*71*), with the *e* value set as 10^−6^.

### Phylogenetic analysis of NUMT sequences

A total of 12 nonreference and three human GRCh38 reference NUMTs were selected for evolutionary analysis. For the nonreference NUMTs, we only included NUMTs with lengths longer than 300 bp and with at least three mismatch base alleles compared to rCRS. For the reference NUMTs, we selected the NUMTs that occurred after the hominin-chimpanzee divergence because we focused on the NUMTs generated within the hominin clade. In addition, we selected the NUMTs with lengths over 300, consistent with the nonreference NUMT analysis. Thus, we lastly obtained three reference NUMTs. To estimate the frequencies of these three reference NUMTs, we first masked these NUMTs in the human GRCh38 reference genome and then extracted reads from each BAM file of 2504 individuals from 1KGP to align to the masked genome. A reference NUMT was considered present in an individual genome if each breakpoint had at least three split reads. We reconstructed the phylogenetic tree using an additional 42 hominin mitochondrial sequences rooted with a chimpanzee mtDNA sequence and estimated the coalescence time of each node in the tree via BEAST v1.8 (*72*) and Tracer v1.5.1 (*73*). The MCMC (Markov chain Monte Carlo) sample was based on a run of 100 million generations sampled every 10,000 steps, with the first 10 million generations regarded as burn-in. We used the HKY+G model of nucleotide substitution without partitioning the whole sequences. A strict clock was used, and the calibration date of archaic DNA (Neanderthals and Denisovans) was according to the previous literature (*74*).

### Enrichment test

To identify regions of nuclear DNA with NUMTs insertion hotspots, our study examined a total of 483 distinct NUMTs distributed throughout the entire genome. To analyze the data, we used two-tailed permutation tests. The UCSC (https://genome.ucsc.edu/) provides downloads of text files containing genomic loci for centromeres, CpG Islands, structural variation, regulatory elements, genomic duplications, microsatellites, retrotransposons, and genes. By using this data, we can calculate the frequency of NUMTs insertion within the 200-bp flanking areas of NUMTs (with 100 bp upstream and 100 bp downstream) for each dataset. The determination of empirical *P* values requires the execution of 1000 permutations, where random positions are generated on a reference complete genome.

To identify the hotspot regions of mtDNA segments from NUMTs, we first calculated the average coverage of mtDNA segments from NUMTs using 50-bp sliding windows with a 10-bp overlap as the empirical value. Subsequently, we randomly selected two points on the mtDNA as breakpoints, ensuring the length and number of NUMTs matched the real data. We then calculated the average coverage using 50-bp sliding windows with a 10-bp overlap, repeating this process 5000 times. To test the significance, we compared each empirical value to the null distribution, merging regions where the *P* value was less than 0.05.

### Comparing call sets with *dinumt*

Given that the same merging strategy for nuclear breakpoints was used in both our approach and *dinumt*, the comparison focused only on the consistency of nuclear breakpoints. When nuclear breakpoints identified through both approaches are within 1000 bp, they are considered as the same NUMTs. Under this criterion, 95 NUMTs reported by *dinumt* were absent from our callset, and 94 of which were on primary scaffolds and retained for comparison. In our short-read data, 73 of these 94 loci showed NUMT-like clusters but did not pass the supported split reads threshold, whereas 21 loci showed no NUMT-supporting cluster.

To further evaluate these loci, we leveraged 1019 long-read genomes from the 1KGP. Among the 94 *dinumt*-unique loci, 40 overlapped at least one long-read sample and were inspected in IGV to assess the presence or absence of clear NUMT insertion signatures. We also identified 56 NUMTs unique to our callset, 19 of which were covered by long-read data and similarly examined in IGV for obvious NUMT insertions.

### Calculating the rate of overlap between NUMTs and introgressed segments

The rate of overlap between NUMTs and introgressed segments was calculated for each individual by the formula *Remp* = (number of NUMTs overlapped with archaic segments)/(total number of NUMTs). The mean rate *Rmean* was calculated for individuals who had NUMTs overlapping introgressed segments.

Permutation tests were performed on the 219 individuals to evaluate the statistical significance of the estimated rates. A total of 5000 simulations were conducted in this procedure. For every individual, a random pairing was performed between NUMT calls and introgressed segments selected from the remaining pool in each simulation. The rates of overlap between these paired sequences were then calculated. The average rate was then calculated from all rates in each simulation. This entire procedure was repeated 5000 times. Subsequently, *Rmean* was compared with the distribution of simulated rates, revealing that none of the 5000 replicates equaled or exceeded the empirical value.

### Tests for recurrent insertion in true introgressed NUMTs

For four true introgressed NUMTs observed in more than one individual, we investigated whether their presence is better explained by a single insertion or by recurrent integration driven by a genomic hotspot. For each locus, we first reconstructed an individual-specific NUMT sequence by deriving a consensus from reads mapping to the NUMT region. Consensus sequences from all carriers of the same locus were then aligned, and pairwise nucleotide identities were calculated and summarized as the mean identity per locus. For NUMTs whose reconstructed sequences overlapped the mitochondrial hypervariable region, alignment positions corresponding to the hypervariable segment were masked, and pairwise identities were recalculated on the masked alignments.

### Selection analysis

To identify NUMTs in regions under selection across 27 populations, we calculated the fixation index (*F*_ST_)–based on PBS (*44*). For each NUMT, the population with the highest allele frequency at that NUMT was designated as the focal population. Population triplets were then constructed for each focal population by selecting a sister group from the same continent and an outgroup from a different continent (for Papuans, East Asian populations were used as the ingroup). The highest PBS value across all possible triplets for each NUMT was identified as a potential signal of selection, and a *z*-score test was then performed using the maximum PBS value of all NUMTs to identify those under significant selection. To determine the genes influenced by putative selective NUMT regions, we extended the NUMT breakpoints by 5 kb upstream and downstream. We identified the nearest gene to each extended region as the potentially affected gene.

To further validate the selection signals identified in Papuans, we applied a haplotype-based test using XP-EHH, with CHB as the reference population. Because NUMTs could not be reliably genotyped, we used the unphased implementation of XP-EHH in selscan v2.0.3 (*75*) with the --xpehh option and the --unphased flag. Genome-wide SNPs and NUMT loci were jointly analyzed, and raw XP-EHH scores were normalized using norm v1.3.0. NUMTs with normalized XP-EHH scores of >2 were considered candidates under selection in Papuans.

### Block-jackknife analysis

To evaluate whether any specific genomic region disproportionately influenced the PBS results, we performed a block-jackknife analysis using two window sizes. The genome was divided into nonoverlapping windows of 500 kb and 1 Mb, and each window covering at least one NUMT was sequentially removed. For each iteration, PBS *z*-scores were recalculated for all NUMTs to assess the stability of the selection signals observed.

### Reconstructing individual introgression calls from Sprime results

The output of the Sprime method is a list of detected introgressed segments and the putative archaic-specific alleles that comprise those segments. We use the output data list to reconstruct the target individuals’ introgression status along their genomes to get specific individual call sets. If the putative archaic-specific alleles occur consecutively on the same haplotype, we categorize the segment formed by these alleles as an introgressed segment in the target individual.

### Gene expression differentiation analysis

We focused on all introgressed and putative introgressed NUMTs present in at least 10 individuals. For each NUMT, samples were divided into two groups based on the presence or absence of the insertion. We examined expression levels of genes located within 200 kb upstream and downstream of each NUMT using RNA-seq data from LCLs of 731 individuals from the 1KGP (MAGE dataset). Differences in gene expression between groups were assessed using the Wilcoxon rank sum test, with significance defined as *P* value < 0.05.

### Allele-specific expression analysis of *RASGRP3*

For the introgressed NUMT *chr2_33M*, we performed allele-specific expression analysis of *RASGRP3* using RNA-seq data from the MAGE dataset. A total of 206 individuals heterozygous at *chr2_33M* were identified from phased genotype data, and biallelic heterozygous SNPs in *RASGRP3* exons were extracted using BCFtools. For each of the 206 heterozygous individuals, allele-specific read counts at exonic heterozygous sites were obtained with GATK (*76*) called ASEReadCounter from the corresponding RNA-seq BAM files, applying minimum mapping and base quality thresholds of 20. Allele counts were summed across informative sites and assigned to the haplotype carrying the NUMT allele or the alternative non-NUMT haplotype and then normalized for sequencing depth to obtain reads per million mapped (RPM). Differences in *RASGRP3* expression between NUMT and non-NUMT haplotypes were evaluated using a paired Wilcoxon signed-rank test.

### Predicting the impact of NUMTs on 3D chromatin structure

We predicted the impact of NUMTs using Akita (*53*), a convolutional neural network for predicting 3D genome folding from DNA sequence alone. We provided Akita with two 1-Mb DNA sequences centered around the NUMT site: one without an NUMT insertion and the other with the NUMT consensus sequence inserted into the middle. After making predictions, we computed the MSE between these two contact matrices to assess their divergence.

### Hi-C processing and visualization for HGSVC3 LCLs

From the HGSVC3 LCLs (Coriell/1000 Genomes), we selected one sample carrying the target NUMT and one sample lacking this NUMT for comparison. Hi-C sequencing reads were processed following the pipeline described in (*77*). Briefly, reads were aligned to the GRCh38 reference genome using Juicer (v1.6) (*78*) with the default BWA-MEM aligner; unmapped, abnormally split, and duplicate reads were removed, and only read pairs with MAPQ ≥ 30 were retained. The resulting high-quality pairs were used to generate contact maps. High-quality pairs were used to build 10-kb contact maps and to perform genome-wide iterative correction (ICE) using cooler (*79*). For locus-level visualization and comparison with Akita predictions, 10-kb contact maps were adaptively coarse-grained, normalized for distance-dependent decay, transformed to log (observed/expected) values, linearly interpolated across missing bins, and smoothed with a 2D Gaussian filter (sigma = 1; width = 5) using Cooltools (*80*).

### Identification of intact mitochondrial reading frames in putative introgressed NUMTs

To evaluate whether introgressed NUMTs retain intact mitochondrial reading frames, each NUMT sequence was aligned to the 13 protein-coding genes of the human mitochondrial genome. We trimmed terminal gaps and excluded alignments containing internal gaps inconsistent with codon structure. Only NUMT-mtDNA pairs whose aligned nucleotide lengths were divisible by three, and that translated without frameshifts or premature stop codons, were classified as carrying an intact mitochondrial reading frame. For NUMTs meeting these criteria, we compared the translated amino acid sequences and quantified synonymous and nonsynonymous differences at the nucleotide level to assess divergence from the mitochondrial reference.
