# Supplementary material for: Introgressed mitochondrial fragments from archaic hominins alter nuclear genome function in modern humans
Source: Sci Adv. 2026 Feb 4;12(6):eaea0706. doi: 10.1126/sciadv.aea0706 (PMC12871456; doi:10.1126/sciadv.aea0706)
Supplement: Supplementary file 1 — Figs. S1 to S12 Legends for tables S1 to S13 [file sciadv.aea0706_sm.pdf]

Science Advances
AAAS

Supplementary Materials for

Introgressed mitochondrial fragments from archaic hominins alter nuclear genome function in modern humans

Qiong Zhu *et al.*

Corresponding author: Hong-Xiang Zheng, zhenghongxiang@fudan.edu.cn; Lu Chen, lu_chen@fudan.edu.cn

Sci. Adv. **12**, eaea0706 (2026)
DOI: 10.1126/sciadv.aea0706

The PDF file includes:

Figs. S1 to S12
Legends for tables S1 to S13

Other Supplementary Material for this manuscript includes the following:

Tables S1 to S13

Supplementary Figures

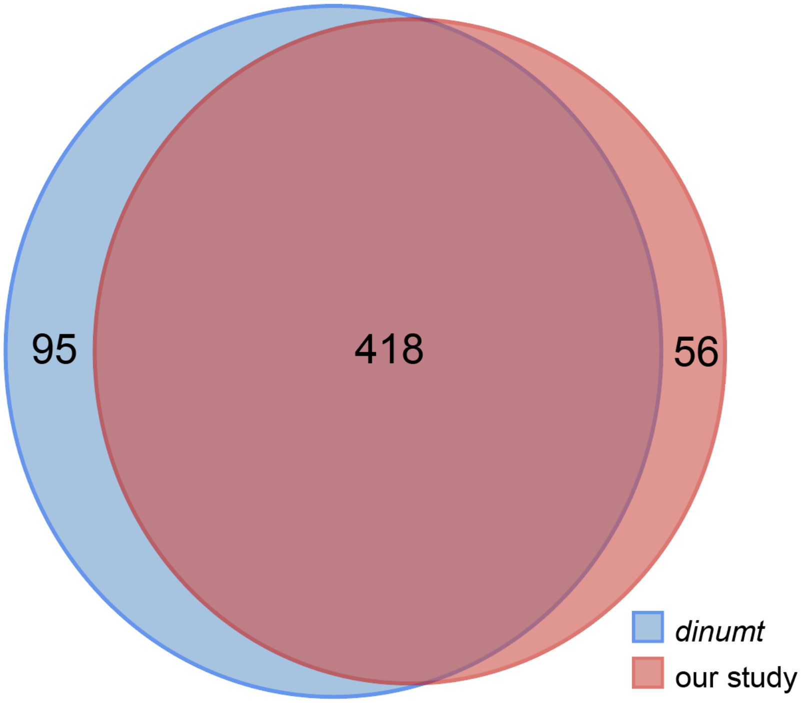

Fig. S1. The Venn diagram of NUMTs identified by two methods. Both methods used the 2,504 unrelated samples from the 1000 Genomes Project aligned to GRCh38. In the Venn diagram, NUMTs identified by *dinumt* are shown in blue, while those identified by our method are shown in red.

A

B

C

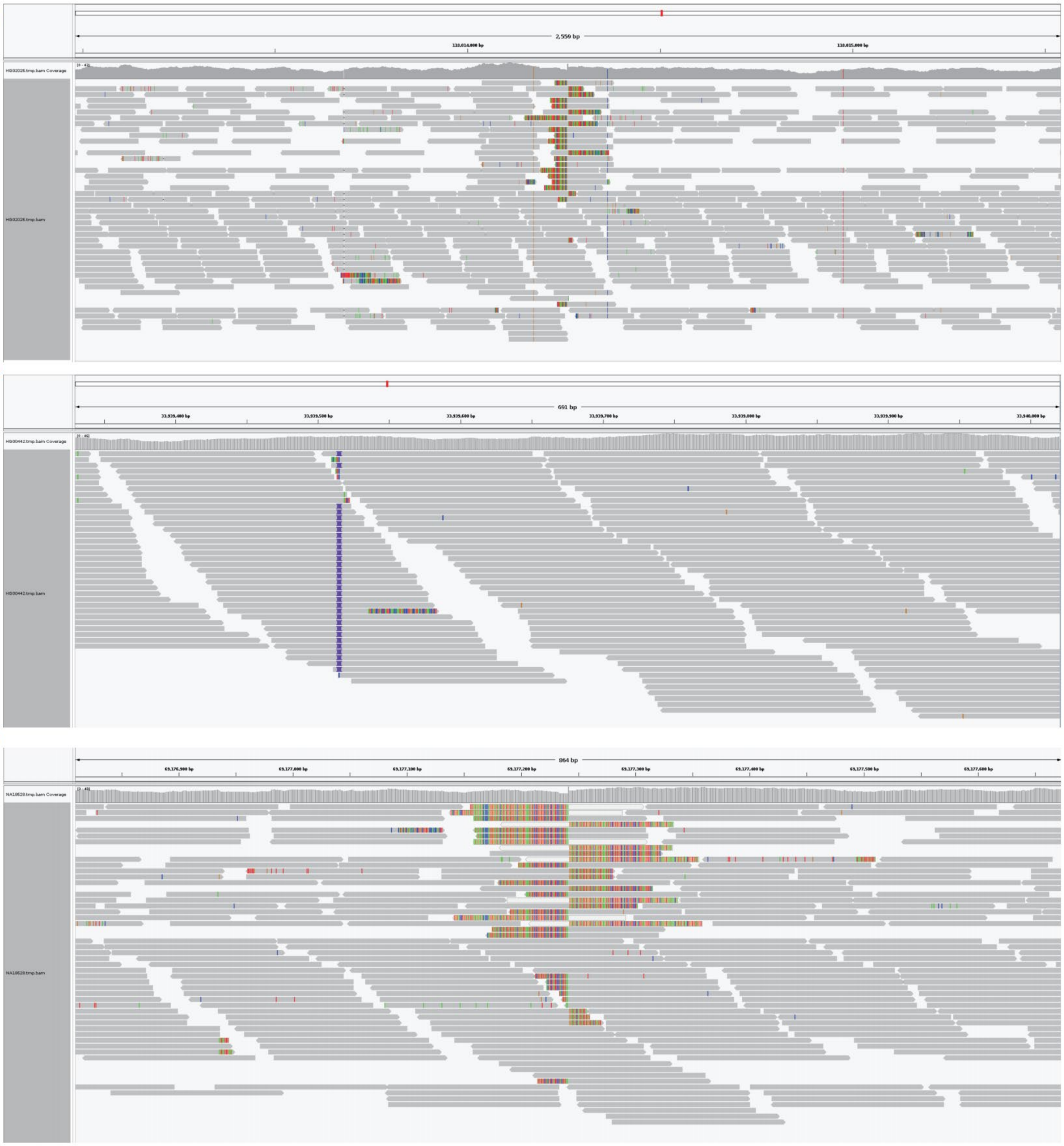

Fig. S2. Representative IGV visualizations of read support for NUMTs uniquely detected by *dinumt* or this study. Colored segments within reads indicate split reads marking putative insertion breakpoints. (A) Example of a *dinumt*-unique site (1/73) not called in this study due to insufficient supporting reads. (B) Example of a *dinumt*-unique site (1/21) not called here because reads do not form a NUMT-supported cluster. (C) Example of a NUMT unique to this study (1/56) with abundant, supported split reads that were not detected by *dinumt*.

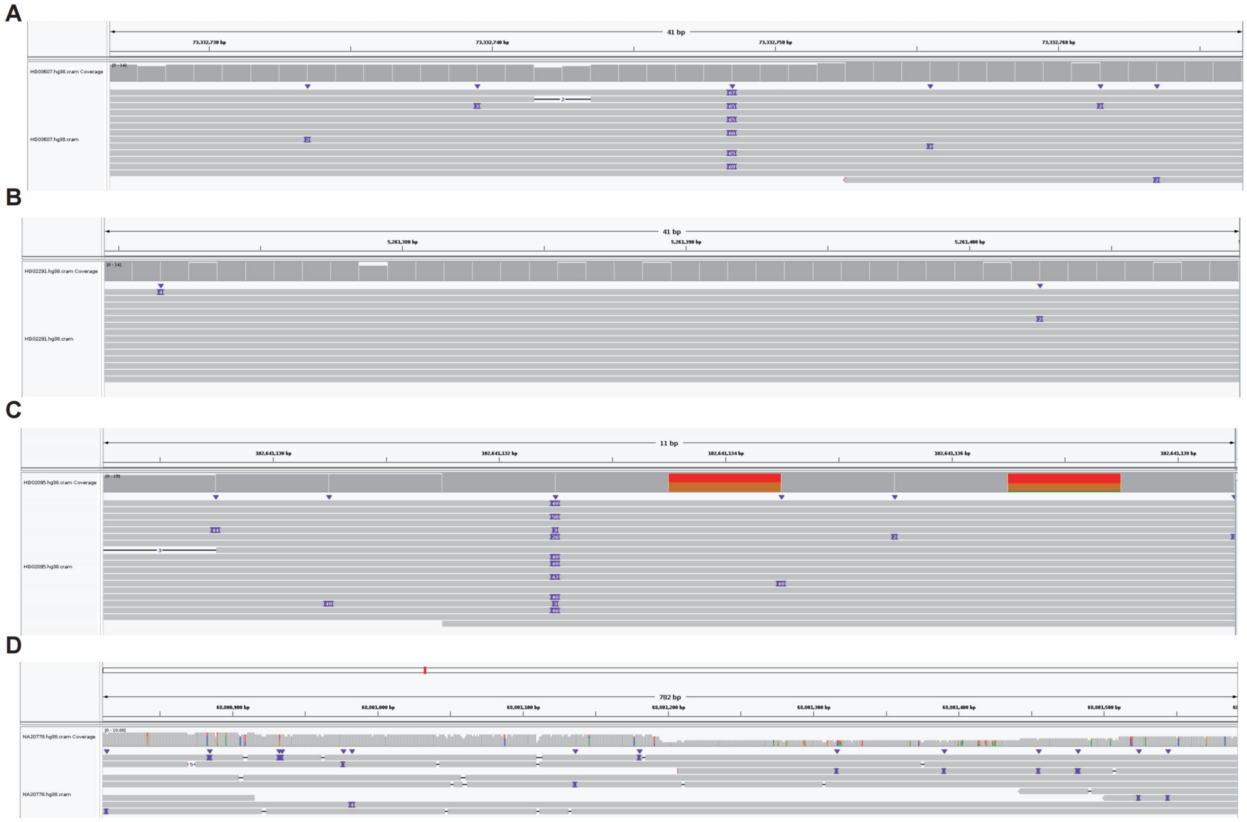

Fig. S3. Long-read evidence for NUMT sites uniquely detected by *dinumt* or this study. Purple blocks within reads mark the inferred NUMT insertion sequence. **(A)** Representative *dinumt*-unique locus with clear NUMT insertion in long-read data; among the 40 *dinumt*-unique loci for which long-read data are available, 20 show this pattern. **(B)** *dinumt*-unique loci without an obvious insertion signal in long-read data (20 of 40 loci). **(C)** NUMT loci uniquely identified by this study that show clear NUMT insertions in long-read data (13 of 19 loci). **(D)** NUMT loci uniquely identified by this study lacked an obvious insertion signal in long-read data (6 of 19 loci).

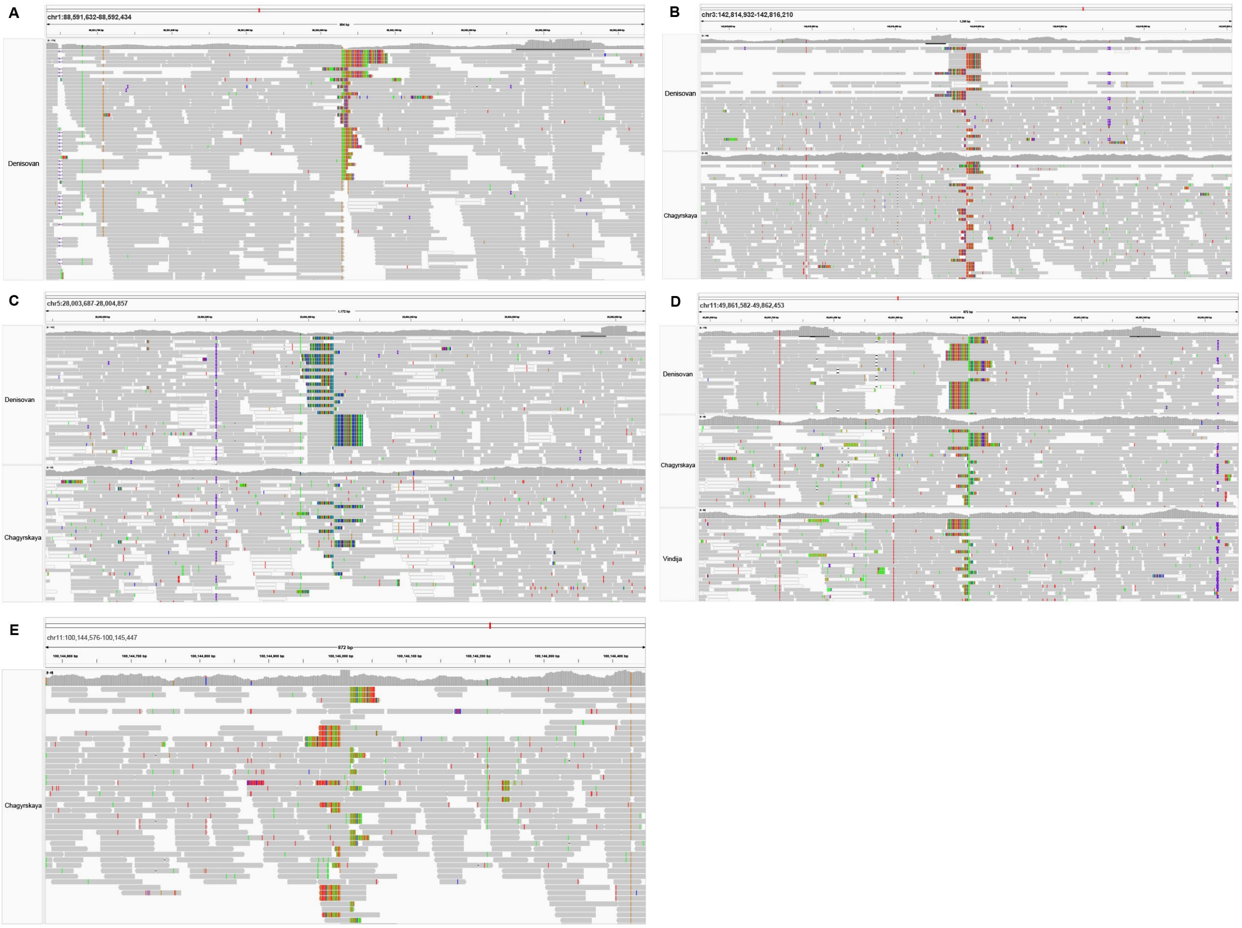

Fig. S4. NUMTs detection in 4 archaic genomes. (A-E) show IGV read-level visualizations at representative NUMT loci in archaic genomes; in each case, junctions between mitochondrial-homologous fragments and flanking nuclear reads define clear NUMT breakpoints. Colored segments within reads indicate split or soft-clipped reads. **(A)** NUMT *chr1_88M* in the Denisovan genome. (B) NUMT *chr3_142M* in Denisovan (top) and Chagyrskaya (bottom). **(C)** NUMTs *chr5_28M* in Denisovan (top) and Chagyrskaya (bottom). **(D)** NUMT *chr11_49M* in Denisovan (top), Chagyrskaya (middle), and Vindija Neanderthal (bottom). **(E)** NUMT *chr11_100M* in the Chagyrskaya genome.

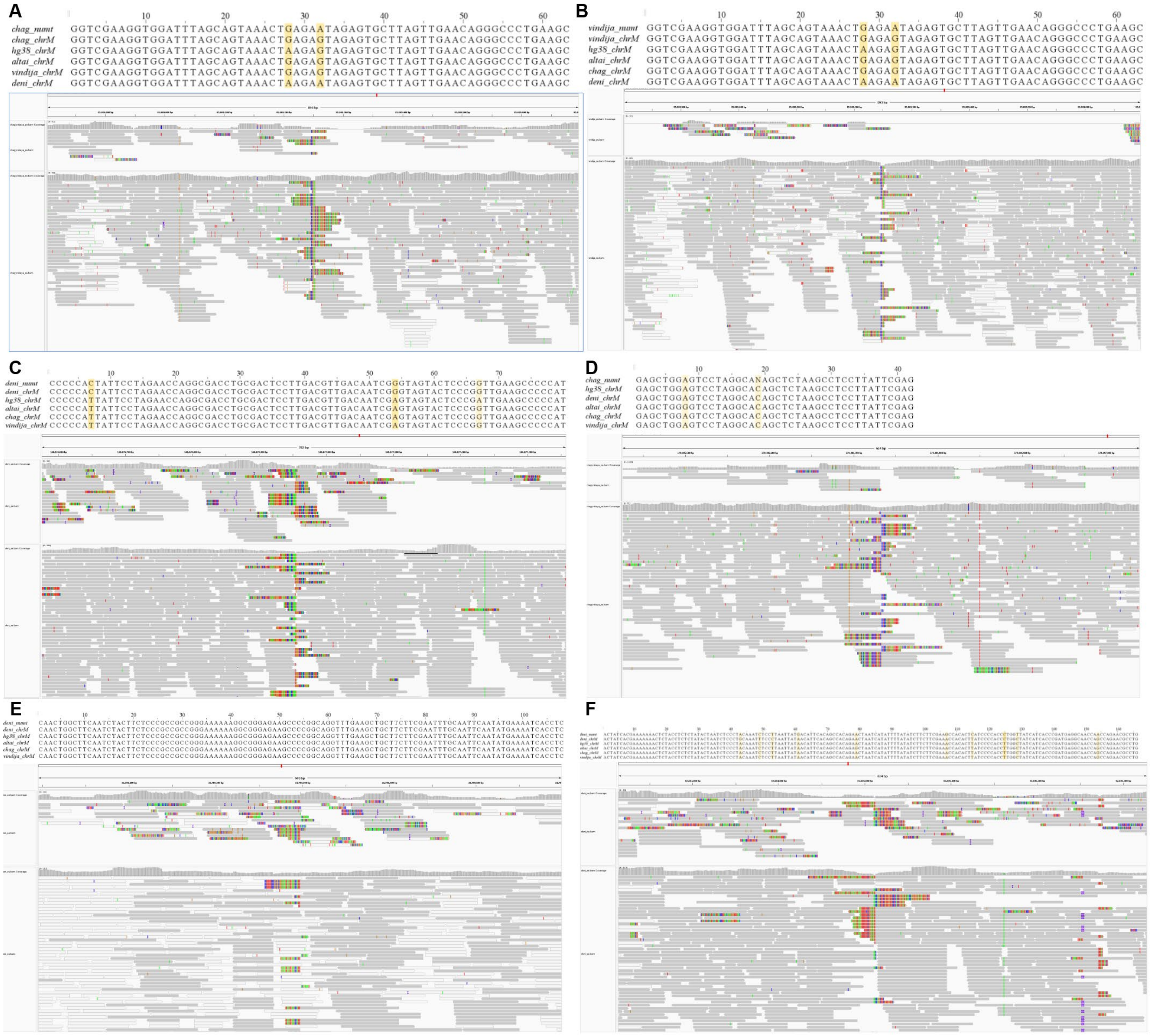

Fig. S5. Sequence alignments and IGV visualizations for NUMTs identified by mtDNA-replacement and manual inspection. Five NUMTs identified by two independent strategies are shown: **(A-C)** correspond to sites detected with the mtDNA-replacement, and **(D-F)** to sites detected by manual inspection. For each locus, the upper panel shows a sequence alignment of the NUMT segment (chag_numt, vindija_numt, or deni_numt) with mtDNA sequences from Denisovan, modern human (GRCh38), and Neanderthal (Altai, Chagyrskaya, and Vindija); nucleotide differences are highlighted in yellow. The corresponding lower IGV panel displays reads mapped across the nuclear insertion site, with colored segments within reads indicating split or soft-clipped fragments at the NUMT breakpoint. **(A)** NUMT *chr9_85M* in Chagyrskaya (chag_numt). **(B)** NUMT *chr9_85M* in Vindija (vindija_numt). **(C)** NUMT *chr2_146M* in Denisovan (deni_numt). **(D)** NUMT *chr4_178M* in Chagyrskaya (chag_numt). **(E)** NUMT *chr21_21M* in Denisovan (deni_numt). **(F)** NUMT *chr4_82M* in Denisovan (deni_numt).

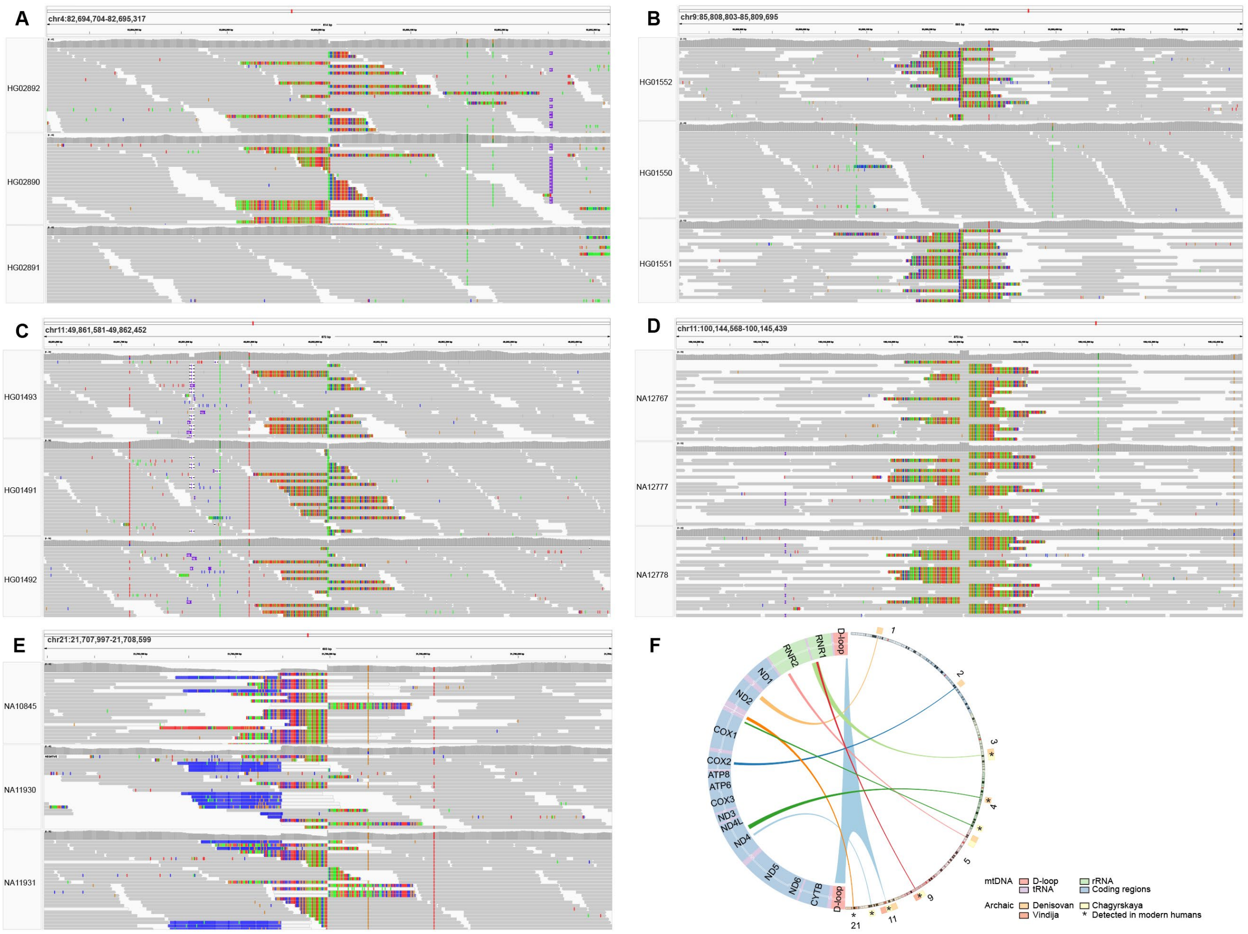

Fig. S6. **Trio-based validation of NUMTs shared between archaic and modern genomes. (A-E)** show IGV read-level views of five NUMTs that are present in both archaic individuals and modern genomes with available trio data. In all trios, a clear NUMT breakpoint is observed in the child (top), and the same breakpoint appears in one or both parents (middle and bottom), consistent with Mendelian inheritance. **(A)** *chr4_82M* (HG02892–HG02890–HG02891). **(B)** *chr9_85M* (HG01552–HG01550–HG01551). **(C)** *chr11_49M* (HG01463–HG01491–HG01492). **(D)** *chr11_100M* (NA12767–NA12777–NA12778). **(E)** *chr21_21M* (NA10845–NA11830–NA11831). **(F)** 10 NUMTs were identified on mtDNA across four archaic genomes, along with their corresponding nuclear insertion sites. * denotes those shared with modern humans.

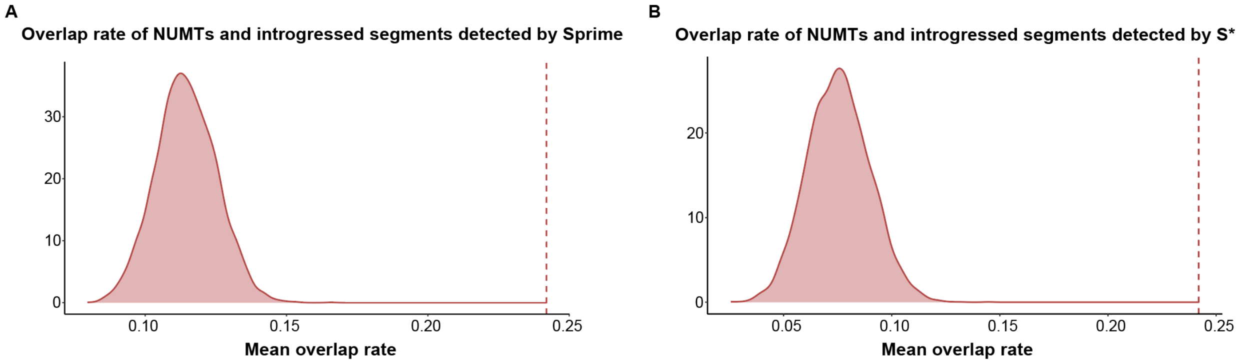

Fig. S7. Enrichment of NUMTs and Neanderthal introgressed segments within the same individual. (A) Distribution of the mean overlap rate between NUMTs and introgressed sequences detected by Sprime, with a dashed line representing empirical data. **(B)** Distribution of the mean overlap rate between NUMTs and introgressed sequences detected by S*, with a dashed line representing empirical data.

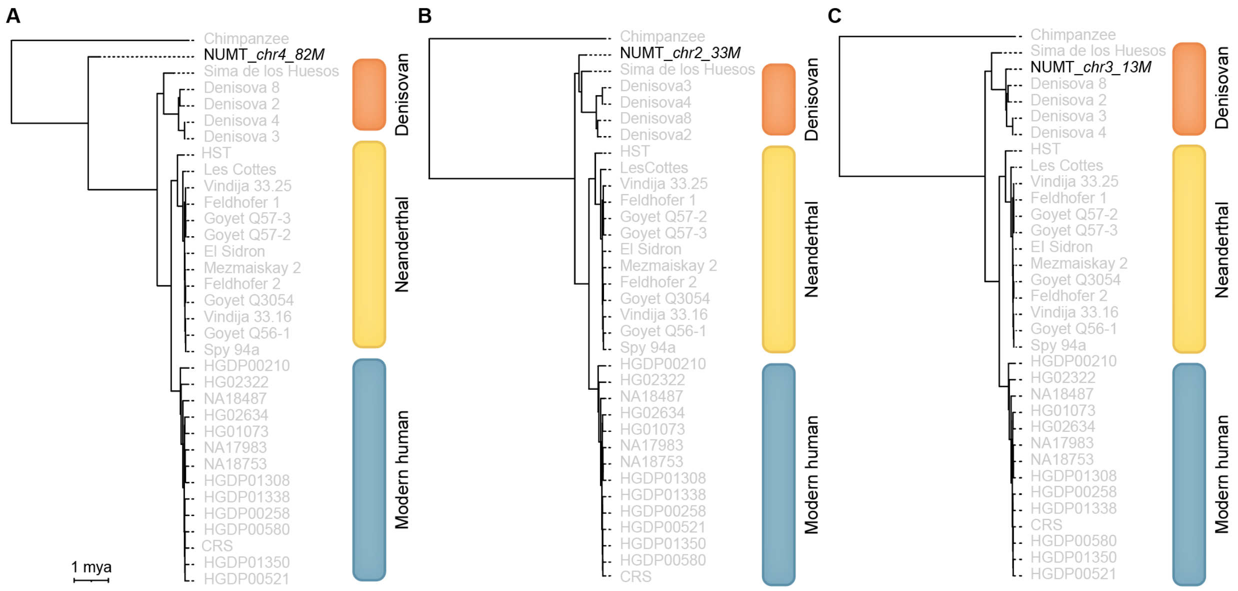

Fig. S8. Bayesian phylogenetic trees of introgressed NUMTs constructed using BEAST. (A-C) Phylogenetic trees for NUMTs (*chr4_82M*, *chr2_33M*, and *chr3_13M*) along with homologous mtDNA sequences from chimpanzee, archaic humans, and modern humans. The orange block represents the Denisovan lineage, the yellow block represents the Neanderthal lineage, and the blue block represents the modern human lineage. The scale bar represents 1 million years ago (mya).

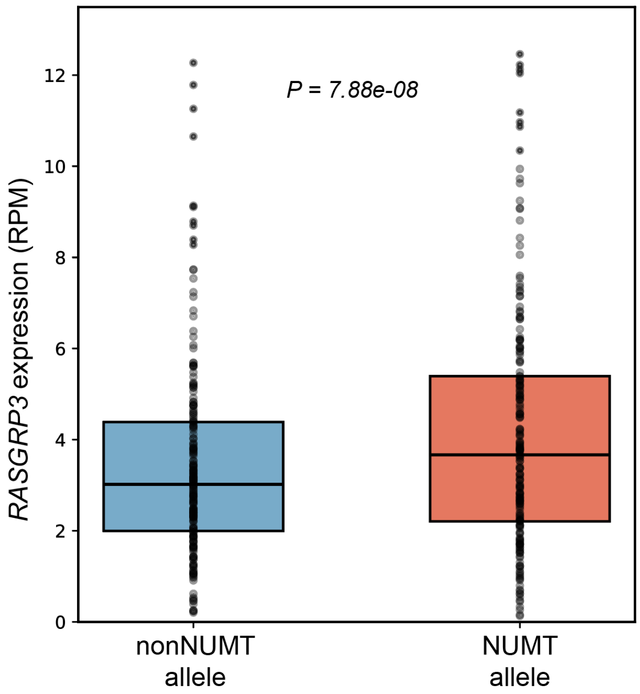

Fig. S9. Allele-specific expression of *RASGRP3* for the introgressed NUMT *chr2_33M*. Boxplots show normalized *RASGRP3* expression (reads per million mapped, RPM) for the chromosome carrying the introgressed NUMT allele (right, “NUMT allele”) and the alternative chromosome without the NUMT (left, “nonNUMT allele”) in 206 individuals heterozygous at *chr2_33M*. Each point represents the expression of the indicated haplotype in one individual. The *P* value shown was obtained using a paired Wilcoxon signed-rank test.

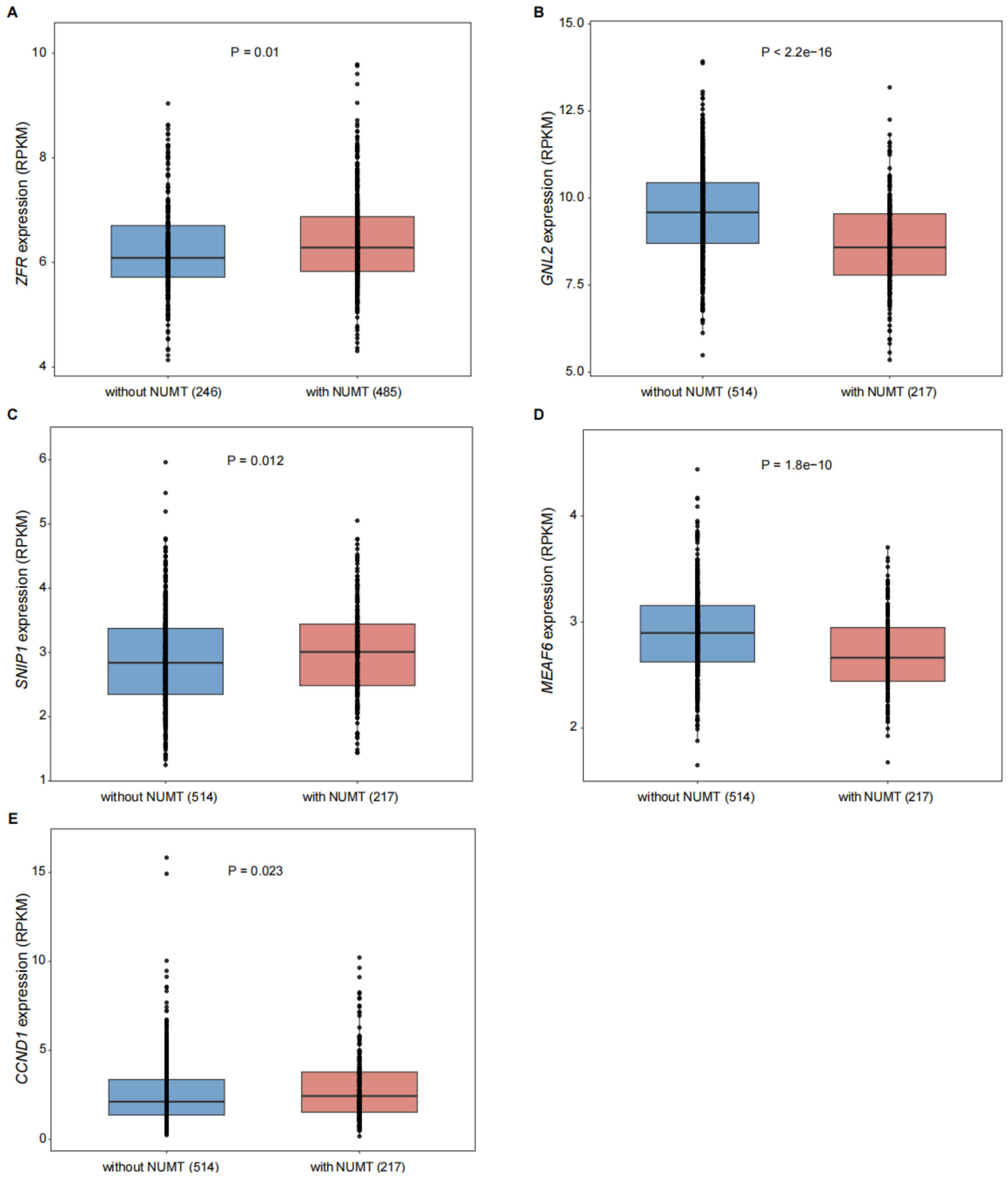

Fig. S10. Gene expression in individuals with and without the putative introgressed NUMTs. Boxplots show expression levels (RPKM) of genes associated with two putative introgressed NUMTs. Individuals are grouped based on the presence or absence of the corresponding NUMT insertion. The Wilcoxon rank-sum test calculated *P* values. **(A)** Expression of *ZFR* in individuals with or without the NUMT *chr5_32M*. **(B–D)** Expression of genes associated with the NUMT *chr1_37M*, including **(B)** *GNL2*, **(C)** *SNIP1*, **(D)** *MEAF6*, and **(E)** *CCND1*. *P*-values were calculated using the Wilcoxon rank-sum test. Sample sizes are indicated in parentheses.

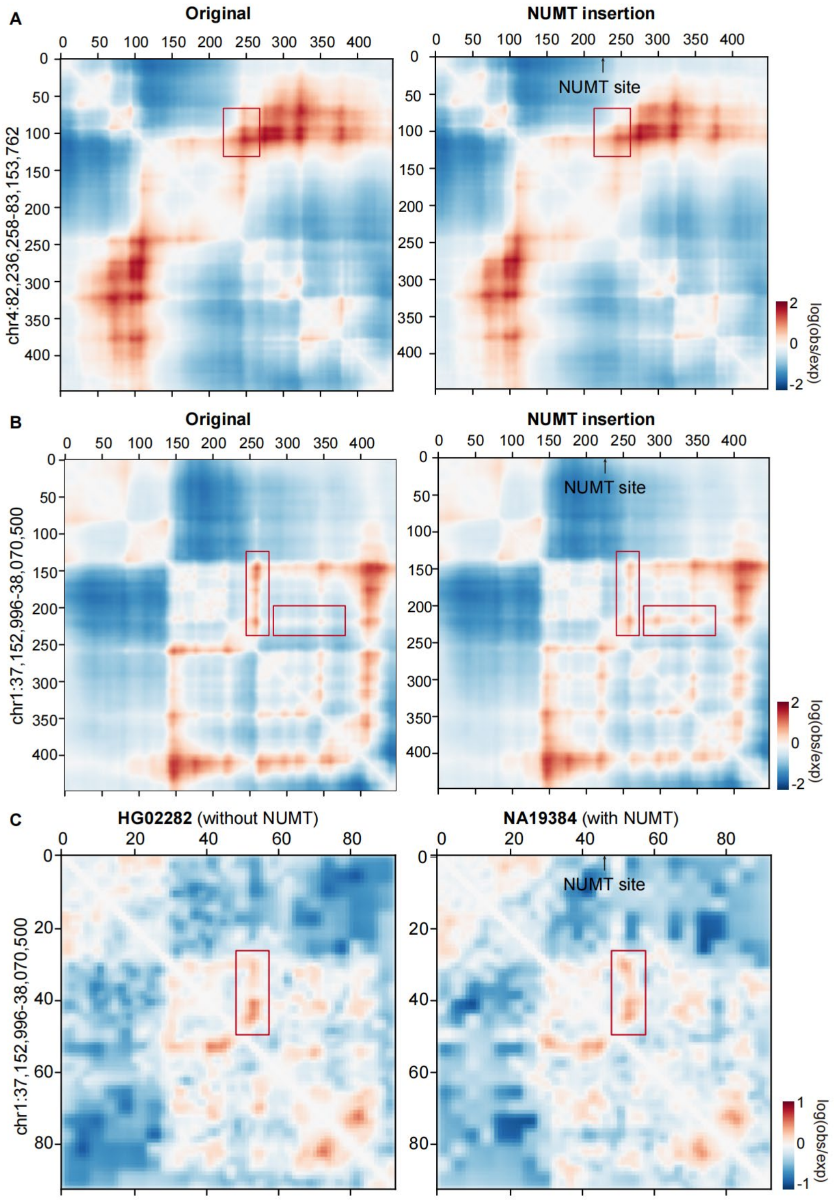

Fig. S11. 3D genome contact matrices from predicted software Akita and experimental data. (A-B) Contact matrices were generated using the Akita deep learning model. The black arrow marks the NUMT insertion site. Red boxes highlight the genomic region where contact frequencies are altered following the insertion. The color scale indicates log(obs/exp) contact frequency. **A:** Locus chr4:82,236,258–83,153,762 without (left) and with (right) a confirmed introgressed NUMT insertion *chr4_82M*. The red box corresponds to the region harboring *SCD5* and *HNRNPD*; **B:** Locus chr1:37,152,996–38,070,500 without (left) and with (right) a putative introgressed NUMT *chr1_37M* insertion. The red box corresponds to the region harboring *GNL2*, *SNIP1*, and *MEAF6*, genes whose expression was shown to be affected in Fig. S10. **(C)** Observed chromatin contact at the chr1:37,152,996–38,070,500 locus for HG02282 (without NUMT; left) and NA19384 (with NUMT *chr1_37M*; right) at 10 kb resolution. The red box highlights the region showing altered contact frequencies after the insertion, and the arrow indicates the NUMT insertion site. The color scale indicates log(obs/exp) contact frequency.

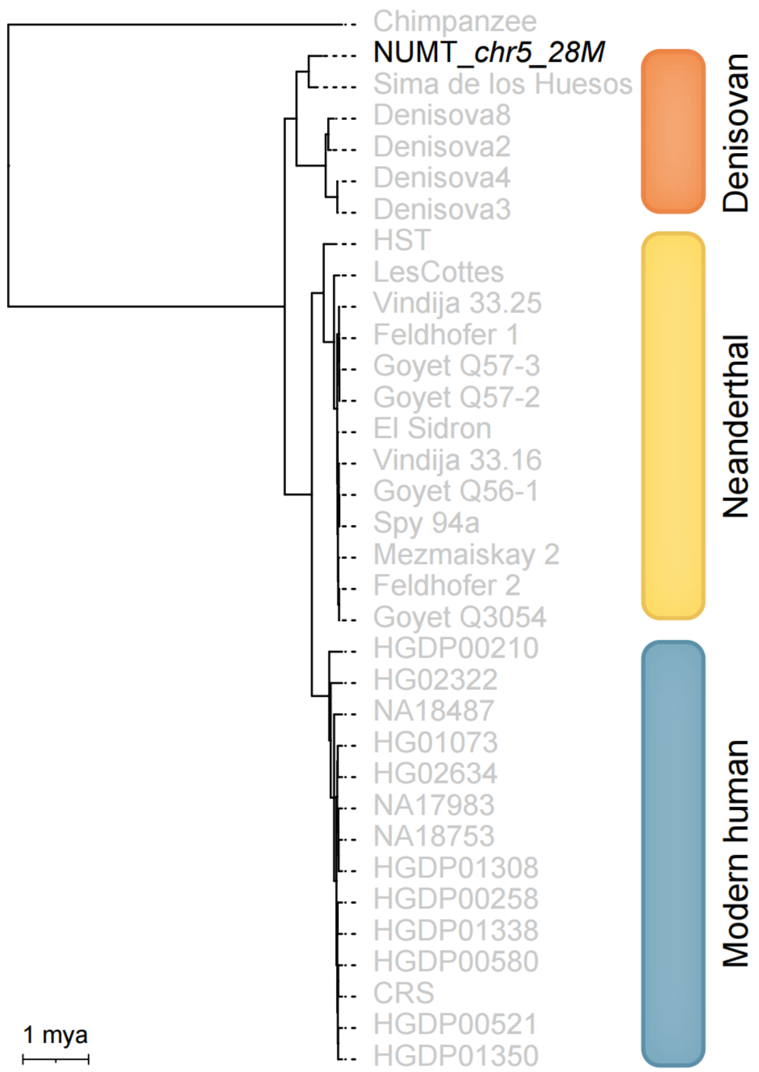

Fig. S12. Bayesian phylogenetic trees of NUMT *chr5_28M* constructed using BEAST. Phylogenetic trees for NUMT *chr5_28M* along with homologous mtDNA sequences from chimpanzee, archaic humans, and modern humans. The orange block represents the Denisovan lineage, the yellow block represents the Neanderthal lineage, and the blue block represents the modern human lineage. The scale bar represents 1 million years ago (mya).

Supplementary Tables
(separate Excel file)

Table S1: All samples used for analysis.
Table S2: 15 Papua New Guinea samples used for analysis.
Table S3: NUMTs detected in modern humans.
Table S4: Number of NUMTs across diverse modern populations.
Table S5: Insertion support for unique NUMTs from *dinumt* and this study in long-read sequencing data.
Table S6: Pairwise t-test comparisons for the distribution of NUMTs per individual among different populations.
Table S7: NUMTs overlapped with Neanderthal segments identified by Sprime and S* in 1KGP individuals.
Table S8: Evidence against recurrent independent insertions of high-confidence introgressed NUMTs.
Table S9: NUMTs overlapped with Denisovan segments identified by Sprime in Papuans.
Table S10: Comprehensive summary of the five best-supported introgressed NUMTs.
Table S11: Selection analysis of NUMTs across populations.
Table S12: Sample groups from MAGE data for gene expression differentiation analysis in introgressed and putative introgressed NUMTs identified in 1KGP.
Table S13: Intact mitochondrial reading frames among putative introgressed NUMTs.
